# GDF15 and its receptors as pathways mediating smoking related weight change

**DOI:** 10.1016/j.ebiom.2025.105806

**Published:** 2025-06-16

**Authors:** Alexander C. Tinworth, Andri Iona, Pang Yao, Iona Y. Millwood, Hannah Fry, Jonathan Clarke, Baihan Wang, Mohsen Mazidi, Christiana Kartsonaki, Robin G. Walters, Huaidong Du, Canqing Yu, Yiping Chen, Dianjianyi Sun, Ling Yang, Dan Valle Schmidt, Jun Lv, Daniel Avery, Liming Li, Derrick A. Bennett, Richard Peto, Robert Clarke, Fiona Bragg, Zhengming Chen, China Kadoorie Biobank Collaborative Group, Junshi Chen, Zhengming Chen, Robert Clarke, Rory Collins, Liming Li, Jun Lv, Richard Peto, Robin Walters, Daniel Avery, Maxim Bernard, Derrick Bennett, Ruth Boxall, Ka Hung Chan, Yiping Chen, Charlotte Clarke, Jonathan Clarke, Huaidong Du, Ahmed Edris Mohamed, Hannah Fry, Simon Gilbert, Pek Kei Im, Andri Iona, Maria Kakkoura, Christiana Kartsonaki, Hubert Lam, Kuang Lin, James Liu, Mohsen Mazidi, Iona Millwood, Sam Morris, Qunhua Nie, Alfred Pozarickij, Maryam Rahmati, Paul Ryder, Dan Schmidt, Becky Stevens, Iain Turnbull, Baihan Wang, Lin Wang, Neil Wright, Ling Yang, Xiaoming Yang, Pang Yao, Xiao Han, Can Hou, Qingmei Xia, Chao Liu, Pei Pei, Dianjanyi Sun, Canqing Yu, Lang Pan, Zengchang Pang, Ruqin Gao, Shanpeng Li, Haiping Duan, Shaojie Wang, Yongmei Liu, Ranran Du, Yajing Zang, Liang Cheng, Xiaocao Tian, Hua Zhang, Yaoming Zhai, Feng Ning, Xiaohui Sun, Feifei Li, Silu Lv, Junzheng Wang, Wei Hou, Wei Sun, Shichun Yan, Xiaoming Cui, Chi Wang, Zhenyuan Wu, Yanjie Li, Quan Kang, Huiming Luo, Tingting Ou, Xiangyang Zheng, Zhendong Guo, Shukuan Wu, Yilei Li, Huimei Li, Ming Wu, Yonglin Zhou, Jinyi Zhou, Ran Tao, Jie Yang, Jian Su, Fang Liu, Jun Zhang, Yihe Hu, Yan Lu, Liangcai Ma, Aiyu Tang, Shuo Zhang, Jianrong Jin, Jiangchao Liu, Mei Lin, Zhenzhen Lu, Lifang Zhou, Changping Xie, Jian Lan, Tingping Zhu, Yun Liu, Liuping Wei, Liyuan Zhou, Ningyu Chen, Yulu Qin, Sisi Wang, Xianping Wu, Ningmei Zhang, Xiaofang Chen, Xiaoyu Chang, Mingqiang Yuan, Xia Wu, Xiaofang Chen, Wei Jiang, Jiaqiu Liu, Qiang Sun, Faqing Chen, Xiaolan Ren, Caixia Dong, Hui Zhang, Enke Mao, Xiaoping Wang, Tao Wang, Xi Zhang, Kai Kang, Shixian Feng, Huizi Tian, Lei Fan, XiaoLin Li, Huarong Sun, Pan He, Xukui Zhang, Min Yu, Ruying Hu, Hao Wang, Xiaoyi Zhang, Yuan Cao, Kaixu Xie, Lingli Chen, Dun Shen, Xiaojun Li, Donghui Jin, Li Yin, Huilin Liu, Zhongxi Fu, Xin Xu, Hao Zhang, Jianwei Chen, Yuan Peng, Libo Zhang, Chan Qu

**Affiliations:** a Clinical Trial Service Unit, Nuffield Department of Population Health, University of Oxford, Oxford, UK; b Department of Epidemiology & Biostatistics, School of Public Health, Peking University, Beijing, China; c Peking University Center for Public Health and Epidemic Preparedness and Response, Beijing, China; d Key Laboratory of Epidemiology of Major Diseases (Peking University), Ministry of Education, Beijing, China; e Health Data Research UK Oxford, University of Oxford, UK

**Keywords:** Smoking, Adiposity, GDF15, Proteomics, Mediation, Mendelian randomization

## Abstract

**Background:**

Smokers have lower body weight than non-smokers, while smoking cessation results in weight gain. Understanding the mechanisms involved can help identify potential therapeutic targets to enhance smoking cessation.

**Methods:**

We measured plasma levels of growth/differentiation factor 15 (GDF15), a stress-responsive protein, and its two receptors (proto-oncogene tyrosine-protein kinase receptor Ret [RET], GDNF family receptor alpha-like [GFRAL]) among 3936 Chinese adults (mean BMI 24.0 kg/m^2^), using Olink and SomaScan platforms. We assessed associations of individual proteins and GDF15/receptor ratios with smoking and adiposity using linear regression. In two-sample Mendelian randomization (MR) analyses, we used genetic variants for smoking intensity from publicly available GWAS as instruments to assess their causal associations with adiposity and plasma levels of proteins in East Asian and European populations. We further assessed the effects of GDF15 and GDF15/receptor ratios in mediating smoking-related weight change.

**Findings:**

Overall, smokers had significantly lower BMI (23.1 [0.2] kg/m^2^) than never-smokers (24.0 [0.1] kg/m^2^), while former smokers had the highest levels of BMI (24.6 [0.2] kg/m^2^) and other measures of adiposity (e.g., waist circumference, waist/hip ratio, and body fat percentage). In observational analyses, smoking was positively associated with GDF15 and with GDF15/receptor ratios from both platforms, with GDF15 levels increasing steeply with number of cigarettes smoked on the assessment day. In MR analyses, smoking intensity was significantly associated with a reduced BMI in East Asians and with higher GDF15 levels in both East Asian and European populations. SomaScan_GDF15 partially mediated the associations of smoking with all adiposity measures, while Olink_GDF15 mediated the association with body fat percentage. The GDF15/RET ratio more robustly mediated the smoking-adiposity relationships than GDF15 alone in both platforms.

**Interpretation:**

In Chinese adults GDF15 plays a role in mediating smoking-related weight change, and could serve as a therapeutic target to facilitate smoking cessation and minimise cessation-induced weight gain.

**Funding:**

10.13039/501100000274British Heart Foundation, 10.13039/501100000289Cancer Research UK, Chinese Ministry of Science and Technology, 10.13039/501100017647Kadoorie Charitable Foundation, 10.13039/501100000265UK Medical Research Council, 10.13039/501100001809National Natural Science Foundation of China, 10.13039/100010269Wellcome Trust.

**Research in context**

**Evidence before this study**

Smokers have a lower body weight than non-smokers, while smoking cessation is associated with significant weight gain that persists over several years, leading to increased risk of disease that may offset some of the benefits of smoking cessation. Such phenomena continue to impact adversely on smoking cessation efforts and on sustaining smoking habits among smokers in many populations. The biological mechanisms underlying smoking-associated weight changes are still poorly elucidated, but nicotine is thought to play a role in appetite suppressing effects of smoking. We searched PubMed for studies examining nicotine related interventions against smoking cessation induced weight gain using the following search terms: (smoking cessation) AND (weight OR BMI) AND (nicotine replacement therapy OR NRT OR nicotine receptor agonist). We found 35 randomised controlled trials (RCTs) that reported effects of nicotine replacement therapy on weight change, which showed inconsistent and only short-term benefits on weight gain compared to placebo. Growth/differentiation factor 15 (GDF15) has been associated with modest weight loss in humans in RCTs, and has also been found to be higher in smokers compared to non-smokers in observational studies. However, the causal associations of GDF15 with smoking and its role in mediating smoking-related weight change remains unexplored.

**Added value of this study**

In this study, we measured plasma levels of GDF15 and its two receptors, proto-oncogene tyrosine-protein kinase receptor Ret (RET), and GDNF family receptor alpha-like (GFRAL) using two affinity-based proteomics platforms (Olink and SomaScan) in 3936 participants in the prospective China Kadoorie Biobank study. In observational analyses, smokers had significantly lower BMI (23.1 [0.2] kg/m^2^) than never-smokers (24.0 [0.1] kg/m^2^), while former smokers had the highest levels of BMI (24.6 [0.2] kg/m^2^) and other measures of adiposity (e.g., waist circumference, waist/hip ratio, and body fat percentage). Moreover, smoking was positively associated with GDF15 and with GDF15/receptor ratios, with levels of GDF15 increasing, in a dose–response fashion, with number of cigarettes smoked on the day of assessment. Our genetic analyses provided strong support for the causal role of smoking on levels of GDF15 and its receptors in both East Asian and European populations. Furthermore, the ratios of GDF15 with its receptors robustly mediated the smoking-adiposity relationships.

**Implications of all the available evidence**

This study establishes the role of GDF15 and its receptors in mediating smoking-associated weight change. Interventions that promote GDF15 expression, such as metformin, either alone or in conjunction with nicotine replacement therapy, may be a cost-effective strategy to improve smoking cessation rates by limiting cessation-induced weight gain.

## Introduction

Tobacco smoking is a leading cause of morbidity and mortality worldwide, accounting for over 8 million deaths annually.^1^ China is the world's largest consumer of tobacco, with over 1 million smoking-attributable deaths a year, chiefly among men, which will continue to increase without substantial rates of smoking cessation.^2^ One barrier to smoking cessation is concern over weight gain,^3^ while in many populations, particularly among young women, smoking is initiated due to its perceived role in weight control.^4^ Observational studies have consistently reported that smokers have lower mean levels of body mass index (BMI) than non-smokers, while the converse is true for ex-smokers.^5^, ^6^, ^7^ In randomised trials, individuals who quit smoking typically experienced an average weight gain of 5–5.5 kg, or an increase in BMI of ∼2 kg/m^2^ within 5 years,^8^ likely offsetting some of the health benefits of smoking cessation.

The biological mechanisms underlying smoking-related weight changes remain unclear. While nicotine has been implicated,^9^ a review of randomised trials of nicotine replacement therapy (NRT) showed substantial heterogeneity between the individual studies’ findings (*I*^2^ = 81%), with at best only small and short-term (∼6 months) effects on post-cessation weight gain. This highlights the need to identify alternative mechanisms underlying smoking-associated weight change.^10^

Growth/differentiation factor 15 (GDF15) is expressed in epithelial cells of several tissues including the liver, often in response to cellular stress.^11^ Previous observational studies of healthy adults and clinical studies of adults with chronic diseases reported higher plasma levels of GDF15 in current smokers compared to non-smokers.^12^, ^13^, ^14^, ^15^, ^16^ Moreover, in pre-clinical studies, GDF15 has been found to suppress appetite and alter food preferences through its receptors, GDNF family receptor alpha-like (GFRAL), expressed in hindbrain neurons and proto-oncogene tyrosine kinase receptor Ret (RET), expressed in various tissue types including brain and endocrine tissues.^17^, ^18^, ^19^, ^20^ These findings have prompted the hypothesis that GDF15 may mediate smoking-induced weight loss and weight gain following smoking cessation, but further evidence from population studies is needed to clarify its role.

We present findings on GDF15 and its two receptors (RET and GFRAL) with smoking and adiposity in ∼4000 participants from the prospective China Kadoorie Biobank (CKB). The main aims of this study are to: (i) examine differences in levels of adiposity between current smokers and non-smokers; (ii) assess associations between smoking and plasma concentrations of GDF15, RET, GFRAL, and ratios of GDF15 with GFRAL and RET; (iii) assess the causal relevance of these associations using Mendelian Randomization (MR) approaches; and (iv) explore whether GDF15 may mediate smoking-related differences in levels of adiposity in Chinese adults.

## Methods

### Study population

Details of the CKB study design, methods and participants have been described previously.^21^ Briefly, CKB is a prospective cohort of 512,724 adults aged 30–79, recruited between 2004 and 2008 from 10 geographically diverse regions across China. At baseline, participants completed an interviewer-administrated, laptop-based questionnaire on socio-demographics (including sex), lifestyle factors, medical history, and medication use. Physical measurements, including anthropometry and blood pressure, were taken by trained technicians using standard and regularly calibrated instruments. Each participant provided a 10-mL non-fasting blood sample, with time since last meal recorded, for long-term storage in liquid nitrogen tanks for future assays.

Health outcomes were monitored by electronic linkage, using each participant's unique national identification number, to mortality and morbidity registries and with national health insurance records.^21^ All causes of death and incident diseases were coded according to the International Classification of Diseases, 10th Revision (ICD-10).

Ethical approvals were granted and maintained by the relevant institutional ethical research communities in the UK (Oxford Tropical Research Ethics Committee) and China (China CDC, Chinese Academy of Medical Sciences and Peking University). All participants provided written informed consent.

### Assessment of smoking status

Smoking information collected included frequency, type and amount of tobacco smoked currently and in the past, in addition to duration of smoking and age at smoking initiation. Among current smokers, information was collected on the number of cigarettes (or equivalent) smoked on the assessment day. Former smokers were additionally asked about their main reason for quitting. Former smokers were either grouped with current smokers as “ever-regular smokers” or excluded to investigate “current-regular smokers” independently. Occasional smokers were grouped with never smokers as “never-regular smokers”.

To validate smoking exposure, exhaled carbon monoxide (COex) levels were measured among all participants using a handheld MicroCO metre, CareFusion, UK.^22^

### Anthropometric measurement

As reported previously,^23^ standing and sitting heights were measured using a calibrated stadiometer, while weight and body fat percentage (BF%) were assessed using a body composition analyser (TANITA-TBF-300GS; Tanita). Body mass index (BMI) was calculated by dividing weight in kilogrammes by height in metres squared. Waist circumference (WC) and hip circumference (HC) were measured using a non-stretch tape measure. Waist/hip ratio (WHR) was calculated as the ratio of WC to HC.

### Proteomic assays

Plasma proteome profiles were measured on the stored baseline samples in a nested case-cohort study, consisting of 1951 acute ischaemic heart disease (IHD) cases and a subcohort of 2026 randomly selected participants. The Olink Explore platform assayed 1463 proteins, including GDF15 and RET, in Uppsala, Sweden, and an additional 1460 proteins, including GFRAL, in Boston, USA on the same samples. Plasma levels of 7288 proteins, including GDF15, GFRAL, and RET, from the same samples, were also assayed using the SomaScan v4.1 aptamer-based platform in Colourado, USA. Normalisation was performed by SomaLogic using adaptive normalisation by maximum likelihood (ANML) to an external reference. Further details on the proteomic assays, quality control and initial data processing are described elsewhere.^24^^,^^25^

### Genetic instrument for smoking

A genetic instrument for daily smoking intensity was created for MR (one and two-sample) analyses using data on cigarettes smoked per day among (i) 34,603 ever-regular smokers in CKB; (ii) 108,275 and 183,196 ever-regular smokers of East Asian and European ancestry (excluding UK Biobank [UKB] and 23&Me) respectively from the GWAS & Sequencing Consortium of Alcohol and Nicotine use (GSCAN) consortium.^26^ Each unit increase in smoking intensity represented an increase from one category of daily cigarette smoking to the next (1–5 [ref], 6–15, 16–25, 26–35, 36+ cigarettes per day). Single nucleotide polymorphisms (SNPs) were selected if they were independent (*r*^2^ < 0.1, distance threshold 250 kb), genome-wide significant (*p* < 5 × 10^−8^) and not previously reported to be associated with the study outcomes.^27^ After excluding SNPs that were missing without a suitable proxy (n = 2) in the outcome datasets, the final genetic instruments included 33 SNPs for East Asians (F-statistic: 84.8; 2.6% variance of daily smoking intensity explained), and 42 for Europeans (82.8; 0.6%) (Supplementary Tables S1 and S2).

To assess the validity of the instrumental variables for the two-sample MR analyses, associations with IHD (14,992 cases, 146,214 controls) and lung cancer (4444 cases, 174,282 controls) were assessed using summary statistics from BioBank Japan (BBJ).^28^ For Europeans, summary statistics for IHD (37,672 cases, 419,724 controls) and lung cancer (246 cases, 420,473 controls) were obtained from UKB.^29^

Outcome datasets for BMI were obtained from BBJ (n = 163,835) and the GIANT consortium (n = 806,834) for East Asians and Europeans respectively.^28^^,^^30^ Summary statistics for WC, HC, WHR, and BF% were obtained from CKB (n = 96,124) and UKB.^29^^,^^31^ Summary statistics for plasma levels of GDF15, GFRAL, and RET in East Asians were derived from GWAS conducted on the Olink and SomaScan assays in the CKB proteomic cohort.^32^ In Europeans, Olink protein levels were from UKB, and an additional immunoassay based GDF15 dataset was obtained from the Generation Scotland Cohort.^33^^,^^34^

In CKB, genotype data were available for 100,706 individuals (of whom, 34,603 were ever-regular smokers), including 803,000 genetic variants, with imputation to ∼20 million common and low frequency variants.^35^ Since the data available prevented restriction of the two-sample analyses to smokers, a smoking intensity genetic score was calculated for each individual by summing the product of each SNP's allele count by their GSCAN effect sizes. This was subsequently assessed in ever-regular smokers against lung cancer, IHD, adiposity measures, and the following negative control variables: age, sex, study area, education, and physical activity to ensure robustness of the genetic instrument against potential pleiotropic pathways.

### Statistics

Participants with missing ambient temperature data were excluded (n = 40), leaving 3936 participants for the final analyses. Plasma protein levels and the GDF15-receptor ratios were analysed on a log-2 scale to ensure normality. The ratios were calculated using the formula log (X/Y) = log(X) – log(Y) where X represents GDF15 and Y represents RET or GFRAL. Pearson's correlation coefficients were used to assess associations between the three proteins and ratios within and between both platforms. Baseline characteristics, across fifths of plasma GDF15 levels, in addition to mean levels of adiposity stratified by never, former, or current-regular smoking status, were estimated using estimated marginal means (EMM).

Mean protein levels and adiposity measures by smoking status (current or never-regular) were estimated using EMM across categories of age and protein levels respectively, adjusted for age (linear and square terms), sex, study area (10 groups), fasting time, ambient temperature (linear and squared terms), plate ID (Olink analyses only), and case-subcohort ascertainment, where appropriate. Age groups were <40, 40–49, 50–59, 60–69, and 70+ and protein levels were grouped into fifths. Former smokers (n = 298) were excluded due to their distinct associations with adiposity when compared with current smokers.

Mean protein levels were assessed across four smoking variables: cigarettes smoked on the assessment day (never smoker, smoker but none today, 1–5, 6–10, 11+), on an average day (never regular, <10, 10–19, 20–29, 30+), duration of (<20, 20–29, 30–39, 40+ years) and age at starting (never regular, ≥25, 20–24, and <20 years old) smoking. Estimates were additionally adjusted for education, BMI, alcohol consumption, physical activity, hypertension status, and kidney disease status. X^2^ tests for trend were applied to the regression coefficients across smoking categories. Mean COex levels were assessed across fifths of GDF15, age, and BMI categories (<18.5, 18.5–22.9, 23.0–27.5, 27.6+ kg/m^2^).

In mediation analyses (overall and sex-specific), the effect of current smoking on GDF15, and the effect of GDF15 on adiposity, controlling for smoking status, were assessed using linear regression. Models were adjusted using the same covariates as the models used to estimate EMMs from the protein-smoking variable associations. The indirect effect (i.e., the effect of smoking on adiposity via GDF15) was calculated as the product of smoking-GDF15 and GDF15-adiposity associations, with 95% confidence intervals estimated using nonparametric bootstrapping (1000 replications).^36^ This was repeated for GDF15/RET and GDF15/GFRAL ratios. Mediation analyses were additionally performed using the difference-in-coefficients method, estimating the change in the beta coefficient of the association between current smoking and each adiposity measure after adding GDF15 to the adjusted regression model.

For MR analyses, we assessed likely deviations from three key assumptions in relation to (i) relevance (i.e., the genetic instrument must be associated with the exposure); (ii) independence (i.e., the instrument is not associated with confounders of the exposure-outcome relationship); and (iii) lack of pleiotropy (i.e., the instrument affects the outcome solely through its effect on the exposure). In two-sample MR analyses, estimates were calculated using the random-effects inverse variance-weighted (IVW) method, with the robustness evaluated using the weighted median, MR-Egger, and MR-PRESSO approaches.^37^, ^38^, ^39^ Continuous outcomes were reported as a standard deviation (SD) change, and binary outcomes as the log odds ratio per category higher of the smoking intensity instrument. If MR Egger indicated significant horizontal pleiotropy (*p* < 0.05) was detected, suggesting a potential violation of the third MR assumption, the instrument was retested after excluding outlier SNPs identified by MR-PRESSO. We applied a Bonferroni correction to account for multiple testing with the use of two instruments against two independent sets of outcomes (adiposity measures and protein levels) (i.e., 0.05/4 = 0.013).

One-sample MR analysis examining associations of the genetic score used linear regression for continuous outcomes and logistic regression for IHD (ICD-10: I20–I25) and lung cancer (ICD-10: C33–C34) in smokers, with controls from the population-representative subset of ever-regular smokers. Estimates were adjusted for age (linear and squared terms), sex, study area (10 groups), 11 genetic national principal components, GWAS array, and population subset status (continuous outcomes only).

**Fig. 1:**
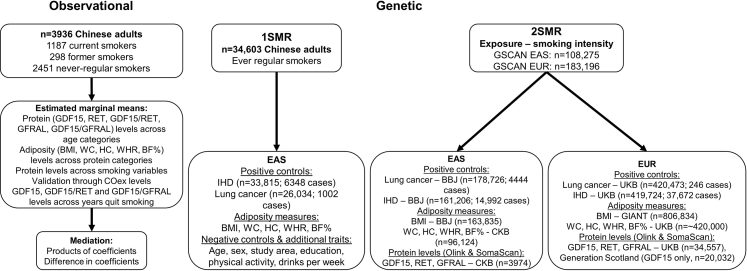
**Flow chart of the study design and analytic approaches.** Abbreviations: BBJ = BioBank Japan; BF% = body fat percentage; BMI = body mass index; CKB = China Kadoorie Biobank; COex = exhaled carbon monoxide; EAS = East Asians; EUR = Europeans; GDF15 = growth/differentiation factor 15; GFRAL = GDNF family receptor alpha-like; GIANT = The Genetic Investigation of ANthropometric Traits; GSCAN = GWAS & Sequencing Consortium of Alcohol and Nicotine use; HC = hip circumference; IHD = ischaemic heart disease; RET = proto-oncogene tyrosine-protein kinase receptor Ret; UKB = UK Biobank; WC = waist circumference; WHR = waist/hip ratio.

Fig. 1 provides an overview of the main analytic approaches used. Analyses were undertaken using R version 4.3.1 using the following packages: ckbplotr, ggplot2, emmeans, TwoSampleMR, and mediation.

### Role of funders

The funders of the study had no role in study design, data collection, data analysis, data interpretation, or writing of this report.

## Results

Among the 3936 participants included, mean age was 57.4 (SD 11.7), mean BMI was 24.0 (3.6) kg/m^2^, and 37.7% (75.1% of males, 5.6% of females) were ever-regular smokers. Mean BMI levels were highest in former smokers (24.6 [0.2] kg/m^2^), followed by never smokers (24.0 [0.1] kg/m^2^), and lowest in current smokers at (23.1 [0.1] kg/m^2^). Similar trends were also observed for other measures of adiposity (Table 1).

**Table 1:** Mean levels of adiposity in 3936 Chinese adults, stratified by smoking status.

Adiposity measure	Mean	Standard error	*p-*value
**Body mass index (kg/m^2^)**			
Never-regular smokers	24.01	0.12	*Reference*
Former smokers	24.57	0.22	0.02
Current smokers	23.08	0.15	4.24 × 10^−8^
**Waist circumference, cm**			
Never-regular smokers	82.25	0.33	*Reference*
Former smokers	83.98	0.62	0.01
Current smokers	80.58	0.41	4.21 × 10^−4^
**Hip circumference, cm**			
Never-regular smokers	91.53	0.23	*Reference*
Former smokers	92.95	0.43	0.002
Current smokers	90.35	0.28	2.75 × 10^−4^
**Waist/hip ratio**			
Never-regular smokers	0.898	0.002	*Reference*
Former smokers	0.901	0.004	0.45
Current smokers	0.891	0.003	0.04
**Body fat percentage, %**			
Never-regular smokers	27.34	0.23	*Reference*
Former smokers	28.18	0.44	0.08
Current smokers	26.44	0.29	0.007

Estimated marginal means for each smoking category were obtained from a linear model, adjusted for age (linear and squared terms), sex, study area (10 regions), education, case-subcohort ascertainment, alcohol drinking and physical activity. *p* values were obtained from the original regression models that informed the mean estimates, using never smokers as the reference group. The cohort was split into 2451 never-regular smokers, 298 former smokers and 1187 current smokers. Data is available for 3932 individuals for body fat percentage.

Using the Olink platform, GDF15 was positively correlated with the GDF15/RET and GDF15/GFRAL ratios (Pearson's correlation: 0.83 and 0.66, respectively), weakly inversely correlated with RET (−0.19), and weakly positively correlated with GFRAL (0.12). Moreover, the GDF15/RET ratio was inversely correlated with RET (−0.71) and weakly with GFRAL (0.06), while the GDF15/GFRAL ratio correlated inversely with GFRAL (−0.67) and weakly with RET (−0.17). These correlations were broadly comparable for SomaScan proteins (Supplementary Figure S1).

Overall, mean plasma Olink_RET levels were higher across fifths of GDF15, but this trend was not apparent based on the SomaScan platform (Table 2). No clear trends were observed in the association of GDF15 with GFRAL. Participants with higher plasma GDF15 levels were, on average, older, more likely to be male, to live in rural areas, and to have regularly smoked. Random plasma glucose and prevalence of diabetes and cancer were positively associated with plasma GDF15 levels. The trends in baseline characteristics by plasma levels of GDF15 were broadly similar between the two platforms, with the exception of more marked negative associations of SomaScan_GDF15 than Olink_GDF15 with adiposity measures.

**Table 2:** Baseline characteristics of participants by fifths of GDF15 concentration measured by Olink and SomaScan in 3936 Chinese adults.

Characteristics^e^	Olink					SomaScan				
	I (n = 788)	II (n = 787)	III (n = 787)	IV (n = 787)	V (n = 787)	I (n = 788)	II (n = 787)	III (n = 787)	IV (n = 787)	V (n = 787)
**Protein concentration, mean (SD)**^a^										
GDF15, NPX/RFU	1.12 (4.23)	1.60 (4.16)	2.05 (4.08)	2.65 (4.12)	4.52 (4.16)	14.1 (13.7)	19.0 (12.8)	23.5 (12.9)	29.2 (12.8)	45.4 (13.2)
GFRAL, NPX/RFU	1.17 (2.73)	1.19 (2.69)	1.14 (2.63)	1.17 (2.66)	1.14 (2.68)	1.12 (6.43)	1.13 (6.43)	1.10 (6.44)	1.34 (6.41)	1.09 (6.62)
RET, NPX/RFU	0.86 (1.10)	0.88 (1.09)	0.92 (1.06)	0.93 (1.08)	0.97 (1.08)	1.41 (0.78)	1.43 (0.73)	1.46 (0.73)	1.42 (0.73)	1.44 (0.75)
**Age, sex, and socioeconomic factors**										
Mean age (SD), years	47.2 (8.6)	53.1 (8.2)	58.7 (8.1)	62.8 (8.2)	65.3 (8.4)	47.1 (8.8)	52.9 (8.3)	58.8 (8.2)	62.6 (8.3)	65.4 (8.6)
Female, %	78.4	64.2	50.4	42.0	34.7	79.0	65.4	54.2	41.0	30.6
≥6 years of education, %	47.9	44.1	43.1	41.4	41.1	46.6	44.8	45.3	40.5	40.5
Annual household income ≥20,000, %	44.1	39.6	40.1	37.8	36.6	42.7	39.6	38.9	39.5	37.6
Urban residency, %	63.7	52.5	47.8	46.0	36.9	71.1	57.5	46.7	43.8	28.3
**Anthropometric and physiological measures, mean (SD)**										
Weight, kg	59.7 (11.6)	60.8 (10.0)	61.2 (9.6)	60.6 (10.2)	59.4 (10.9)	61.4 (11.6)	61.9 (10.0)	61.1 (9.6)	59.6 (10.0)	57.8 (11.0)
Height, cm	159.2 (6.7)	158.9 (5.8)	158.8 (5.6)	158.5 (5.9)	158.5 (6.4)	159.2 (6.8)	159.2 (5.9)	158.7 (5.6)	158.4 (5.9)	158.5 (6.4)
Body mass index, kg/m^2^	23.5 (4.1)	24.0 (3.5)	24.2 (3.4)	24.0 (3.6)	23.5 (3.9)	24.1 (4.1)	24.3 (3.6)	24.2 (3.4)	23.7 (3.6)	22.9 (3.9)
Waist circumference, cm	80.4 (11.5)	82.1 (9.9)	82.7 (9.6)	82.1 (10.1)	81.2 (10.8)	82.2 (11.5)	82.9 (10.0)	82.8 (9.5)	81.0 (10.0)	79.7 (10.9)
Hip circumference, cm	91.0 (7.8)	91.5 (6.8)	91.5 (6.5)	91.0 (6.9)	90.8 (7.4)	92.0 (7.9)	91.9 (6.8)	91.5 (6.5)	90.7 (6.8)	89.8 (7.4)
Body fat percentage, %^b^	26.8 (8.1)	27.9 (7.0)	27.8 (6.8)	27.4 (7.1)	26.4 (7.6)	28.2 (8.1)	28.3 (7.0)	27.9 (6.7)	26.8 (7.0)	25.1 (7.7)
Waist-hip-ratio	0.88 (0.08)	0.90 (0.07)	0.90 (0.07)	0.90 (0.07)	0.89 (0.08)	0.89 (0.08)	0.90 (0.07)	0.90 (0.07)	0.89 (0.07)	0.88 (0.08)
SBP, mmHg	135.5 (26.6)	137.1 (23.0)	139.0 (22.2)	139.8 (23.4)	139.8 (25.0)	139.1 (26.8)	137.9 (23.2)	140.0 (22.2)	137.5 (23.3)	136.9 (25.3)
RPG, mmol/L^c^	5.8 (3.8)	6.1 (3.3)	6.5 (3.2)	6.8 (3.3)	7.4 (3.6)	5.8 (3.8)	6.1 (3.3)	6.5 (3.2)	6.8 (3.3)	7.4 (3.6)
Exhaled carbon monoxide, ppm	5.7 (0.3)	7.6 (0.3)	8.8 (0.3)	8.9 (0.3)	9.3 (0.3)	5.6 (0.3)	7.5 (0.3)	8.6 (0.3)	9.2 (0.3)	9.4 (0.3)
**Lifestyle factors**										
Ever regular smoker, %	33.4	36.6	39.3	44.3	48.5	33.9	36.5	41.2	41.6	48.7
Ever regular alcohol drinker, %	17.0	17.2	18.2	21.1	20.6	16.8	18.4	18.8	20.4	20.0
Physical activity, MET-h/day (SD)	17.6 (13.3)	17.9 (11.5)	18.7 (11.1)	18.1 (11.7)	17 (12.5)	17.6 (14.0)	18.1 (11.6)	18.4 (11.1)	18.0 (11.6)	17.2 (12.6)
**Self-reported disease status, %**										
Diabetes^d^	3.5	6.0	10.1	14.2	19.1	7.0	8.8	9.8	14.3	17.1
Cancer	0.0	0.0	0.7	0.7	1.7	3.8	5.7	11.2	13.2	18.7
Kidney disease	0.6	0.3	1.3	2.2	1.4	0.0	0.6	0.7	0.4	1.4
Hepatitis/Cirrhosis	0.7	1.0	0.6	1.4	1.5	0.1	1.0	1.2	1.7	1.7
Self-rated poor health	5.3	9.2	10.9	12.3	19.5	1.3	0.6	0.6	1.3	1.4

Abbreviations: GDF15 = growth/differentiation factor 15; GFRAL = GDNF family receptor alpha-like; MET = metabolic equivalent of task; NPX = normalised protein expression; RET = proto-oncogene tyrosine-protein kinase receptor Ret; RFU = relative fluorescence units; RPG = random plasma glucose; SBP = systolic blood pressure; SD = standard deviation.

a Estimates for protein levels displayed on a linear scale, with SomaScan RFU further divided by 1000.

b Data available for 3932 participants.

c Data available for 3927 participants.

d Self-reported previously diagnosed or screen detected (RPG ≥7.0 mmol/L with fasting time ≥8 h or RPG ≥11.1 mmol/L with fasting time <8 h or fasting plasma glucose level ≥7.0 mmol/L on subsequent testing) diabetes.

e Adjusted for age (linear and squared terms), sex, study area (10 groups) and case-subcohort ascertainment as appropriate. Protein levels additionally adjusted for fasting time, temperature (linear and squared terms) and plateID (Olink only).

**Fig. 2:**
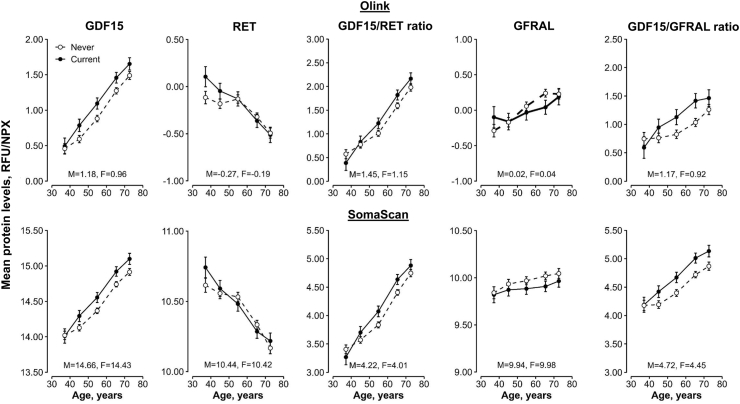
**Associations of age with plasma levels of GDF15, RET and GFRAL and their ratios measured by Olink and SomaScan by smoking status in 3638 Chinese adults**. Former smokers (n = 298) were excluded. Estimated marginal means (95% CI) for each age or sex category among 1187 current and 2451 never-regular smokers were obtained from a linear model adjusted for age (linear and squared terms, where appropriate), sex (where appropriate), study area (10 groups), fasting time, ambient temperature (linear and squared terms), plate ID (Olink analyses only), and case-subcohort ascertainment. The length of the y-axis represents approximately 3 standard deviations of the corresponding plasma protein concentration. NPX values correspond to Olink measurements and RFU values to SomaScan measurements. Abbreviations: CI = confidence interval, F = females; GDF15 = growth/differentiation factor 15; GFRAL = GDNF family receptor alpha-like; M = males; NPX = normalised protein expression; RET = proto-oncogene tyrosine-protein kinase receptor Ret; RFU = relative fluorescence units.

Mean GDF15 levels were higher with increasing age, while RET levels were lower at older ages (Fig. 2). In all but the youngest age group, mean plasma GDF15 levels were higher among current than never-regular smokers and men had, on average, higher GDF15 levels than women. Plasma RET levels were higher among current smokers at ages <50 years, but differed little by smoking status at older ages. These associations translated into higher GDF15/RET ratio levels at older ages, among current smokers at ages over 50, and among men. There was a modest positive association of GFRAL levels with age, but no consistent trend according to smoking status. Levels of the GDF15/GFRAL ratio were higher at older age. These associations were qualitatively similar across the two proteomic panels and between men and women, despite the wider confidence intervals in women due to fewer current smokers (Supplementary Figures S2 & S3).

**Fig. 3:**
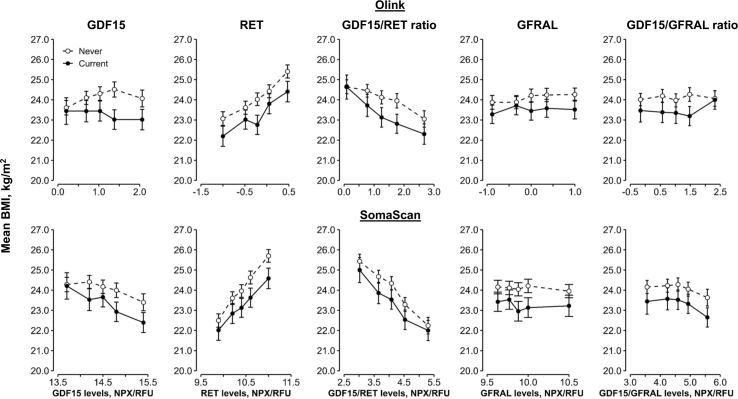
**Associations of plasma levels of GDF15, RET and GFRAL and their ratios measured by Olink and SomaScan with BMI, by smoking status in 3638 Chinese adults**. Former smokers (n = 298) were excluded. Estimated marginal means (95% CI) for each protein fifth among 1187 current and 2451 never-regular smokers were obtained from a linear model adjusted for age (linear and squared terms), sex, study area (10 groups), fasting time, ambient temperature (linear and squared terms), plate ID (Olink analyses only), and case-subcohort ascertainment. The length of the y-axis represents approximately 2 standard deviations of BMI. NPX values correspond to Olink measurements and RFU values to SomaScan measurements. Abbreviations: BMI = body mass index; CI = confidence interval, GDF15 = growth/differentiation factor 15; GFRAL = GDNF family receptor alpha-like; NPX = normalised protein expression; RET = proto-oncogene tyrosine-protein kinase receptor Ret; RFU = relative fluorescence units.

There was an apparent inverse dose–response association between GDF15 levels measured on the SomaScan platform and BMI, but no clear association between GDF15 levels measured on the Olink platform and BMI (Fig. 3). There was a positive, approximately linear, association between RET levels and BMI, and an inverse dose response relationship between GDF15/RET levels and BMI. However, BMI differed little across fifths of GFRAL, or the GDF15/GFRAL ratio. BMI was consistently lower in current compared to never-regular smokers across protein level fifths, overall and in males and females separately (Supplementary Figures S3 & S4). Associations of levels of each protein with WC, HC, WHR, and BF% were broadly comparable to those with BMI (Supplementary Figures S5–S9). These associations were similar between the two assay panels.

**Fig. 4:**
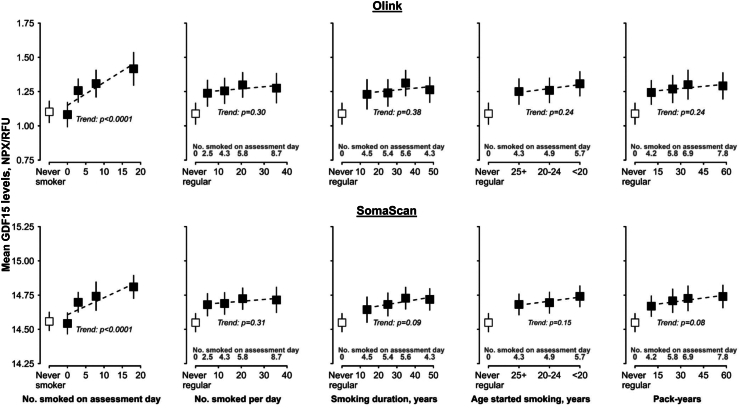
**Associations of smoking intensity with plasma levels of GDF15 measured by Olink and SomaScan in 3638 Chinese adults**. Former smokers (n = 298) were excluded. Estimated marginal means (95% CI) for each smoking category among 1187 current and 2451 never-regular smokers were obtained from a linear model adjusted for age (linear and squared terms), sex, study area (10 groups), fasting time, ambient temperature (linear and squared terms), plate ID (Olink analyses only), BMI, alcohol, physical activity, hypertension status, kidney disease status and case-subcohort ascertainment. Tests for trend include only current smokers. NPX values correspond to Olink measurements and RFU values to SomaScan measurements. Abbreviations: GDF15 = growth/differentiation factor 15; NPX = normalised protein expression; RFU = relative fluorescence units.

Among current smokers, there was a positive linear association between number of cigarettes smoked on the assessment day and mean GDF15 levels (*p*_*trend*_ < 0.0001 for Olink and SomaScan panels) (Fig. 4). Weaker, non-significant, associations were observed between other indicators of frequency or intensity of smoking, including cigarettes smoked per day, smoking duration, age at starting smoking, pack-years, and GDF15. Smoking duration (*p*_*trend*_ = 0.03 and 0.01 for Olink and SomaScan respectively) and pack-years (*p*_*trend*_ = 0.02 for SomaScan) were inversely linearly associated with RET levels, but there were no associations of the other smoking variables studied with RET (Supplementary Figure S10). Consequently, number of cigarettes smoked on the assessment day (*p*_*trend*_ < 0.0001 for Olink and SomaScan panels), smoking duration (*p*_*trend*_ = 0.03 and 0.004, respectively) and pack-years (*p*_*trend*_ = 0.24 and 0.006, respectively) were positively associated with GDF15/RET levels (Supplementary Figure S11). Mean Olink_GFRAL levels were lower with higher number of cigarettes smoked on the assessment day (*p*_*trend*_ = 0.04) and smoking duration (*p*_*trend*_ = 0.001), but no apparent associations were observed for cigarettes smoked per day, age at starting smoking, or pack-years (Supplementary Figure S12). The findings for GDF15/GFRAL ratio levels were similar to those for GDF15 and the GDF15/RET ratio, with the exception of there being no significant trend for pack-years (Supplementary Figure S13). For all four protein measures, with the exception of some difference in statistical significance, their associations with smoking intensity/frequency were broadly consistent across the two panels.

**Fig. 5:**
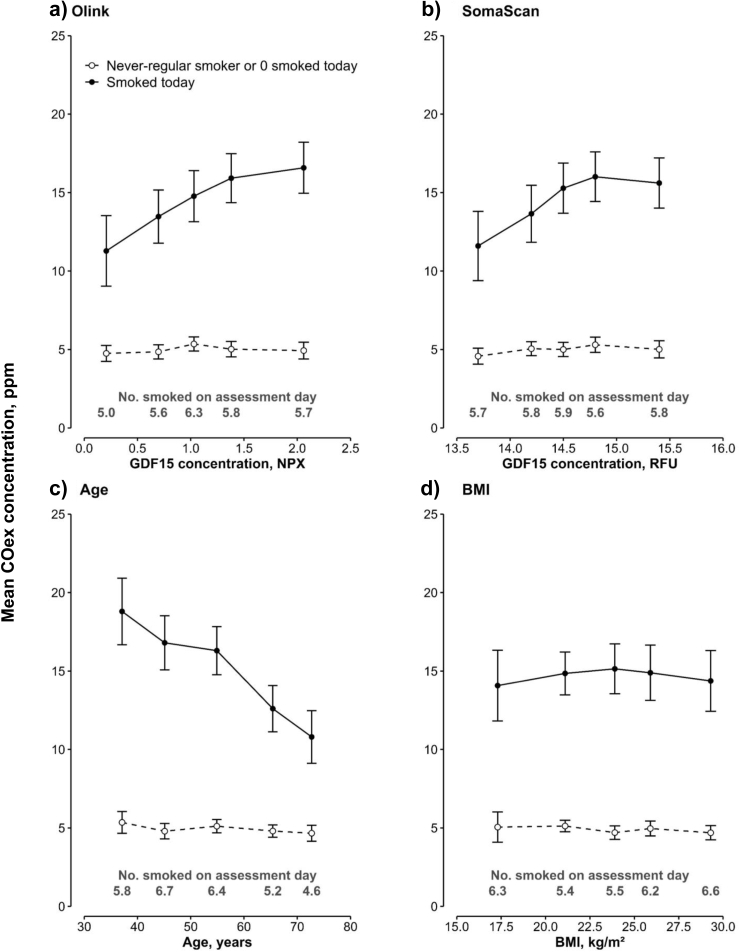
**Associations of exhaled carbon monoxide levels with GDF15 measured by Olink and SomaScan, age and BMI, by smoking status in 3936 Chinese adults**. Estimated marginal means (95% CI) for each category among 1035 individuals who smoked on the assessment day and 2901 who did not were obtained from a linear model. c) and d) were adjusted for age (linear and squared terms, where appropriate), sex, study area (10 groups) and case-subcohort ascertainment, a) and b) were additionally adjusted for fasting time, ambient temperature (linear and squared terms) and plate ID (Olink only). Abbreviations: BMI = body mass index; COex = exhaled carbon monoxide; GDF15 = growth/differentiation factor 15; ppm = parts per million.

Among individuals who smoked on the assessment day, mean COex levels were higher in individuals with higher GDF15 levels (Fig. 5). Furthermore, there was an inverse association of mean COex levels with age, potentially indicative of fewer cigarettes smoked on the assessment day, but no association with BMI. Among individuals who did not smoke on the assessment day, there were no associations of COex with GDF15, age, or BMI. In former smokers, GDF15 levels were lower in those with short quitting durations before appearing to be higher, though non-significantly, with longer durations of quitting (Supplementary Figure S14).

### Mendelian Randomization

**Fig. 6:**
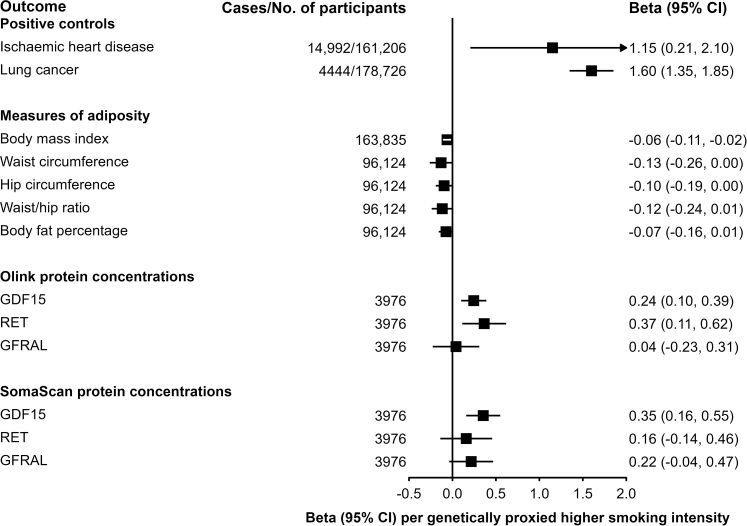
**Associations of genetically-proxied daily smoking intensity with ischaemic heart disease, lung cancer, measures of adiposity and plasma levels of GDF15, RET and GFRAL in 2-sample MR analyses among East Asians**. Estimates reflect a 1-category higher in category of cigarettes smoked per day, with 1–5 cigarettes as the baseline. For ischaemic heart disease and lung cancer, the beta represents the log odds ratio per category higher in genetically-proxied daily smoking intensity. For continuous outcomes, the beta represents the standard deviation change per category higher. Significant associations with evidence of horizontal pleiotropy (Egger test *p* < 0.05) that remained significant after MR-PRESSO adjustments were re-evaluated by repeating the Egger test after excluding MR-PRESSO identified outlier variants. The Bonferroni corrected *p*-value was 0.013. Abbreviations: BMI = body mass index; GDF15 = growth/differentiation factor 15; GFRAL = GDNF family receptor alpha-like; RET = proto-oncogene tyrosine-protein kinase receptor Ret.

In two-sample MR analyses in East Asians, one category higher in genetically-proxied smoking intensity was associated with lower levels of BMI (β = −0.06 [−0.02, −0.11], *p* = 0.009 [random-effects IVW]) and WC (β = −0.13 [−0.00, −0.26], *p* = 0.048 [random-effects IVW]), though WC did not pass correction for multiple testing. Other measures of adiposity showed directionally consistent but non-significant associations (Fig. 6). Furthermore, genetically-proxied smoking intensity was also associated with higher Olink_GDF15 (β = 0.24 [95% CI 0.10, 0.39], *p* = 0.0009 [random-effects IVW]) and RET (β = 0.37 [0.11, 0.62], *p* = 0.005 [random-effects IVW]), but not GFRAL (β = 0.04 [−0.23, 0.31], *p* = 0.77 [random-effects IVW]), with no evidence for horizontal pleiotropy for GDF15 (*p* = 0.64 [MR-Egger]) or RET (*p* = 0.11 [MR-Egger]) (Supplementary Table S3). For SomaScan_GDF15 and GFRAL, these associations were consistent, but no association was observed with RET (β = 0.16, [−0.14, 0.46], *p* = 0.30 [random-effects IVW]).

Expectedly, the genetic instrument for smoking was associated with a higher risk of IHD (OR = 3.16 [1.23–8.17], *p* = 0.02 [random-effects IVW]) and lung cancer (OR = 4.95, [3.86, 6.36], *p* < 0.0001 [random-effects IVW]). However, significant horizontal pleiotropy was observed for IHD, BMI, and WC (*p* < 0.05 [MR-Egger]), but these became non-significant after excluding MR-PRESSO-identified outliers, except for WC, where the PRESSO-adjusted estimate was no longer significant. Horizontal pleiotropy was additionally detected for the SomaScan_GDF15 association (*p* = 0.009 [MR-Egger]), although no outlier variants were identified by MR-PRESSO. Across all analyses, the findings were directionally consistent with the weighted median estimates.

In Europeans, associations were consistent with East Asians for GDF15, GFRAL, and lung cancer (Supplementary Table S4). However, the instrument was not associated with IHD, adiposity measures, or RET. Horizontal pleiotropy was detected for the Olink_GDF15 association (*p* < 0.02 [MR-Egger]), but no outlier variants were identified.

In one-sample MR analyses in CKB, the weighted genetic score was associated with reduced levels of BMI (β = −0.36, [−0.21, −0.50], *p* < 0.0001 [linear regression]) and all other measures of adiposity, in addition to a higher risk of lung cancer (OR = 1.51, [95% CI 1.13–2.01], *p =* 0.005 [logistic regression]) (Supplementary Table S5). Furthermore, the weighted score was not significantly associated with any of the negative controls implausibly caused by smoking. However, there was no significant association with risk of IHD (OR = 1.03 [0.90–1.19], *p* = 0.64 [logistic regression]) and a significant association with lower weekly alcohol consumption (β = −133.6, [−124.3, −142.9], *p* < 0.0001 [linear regression]).

### Mediation analysis

Using the products of coefficients approach, SomaScan_GDF15 appeared to significantly mediate some of the association between smoking and BMI (indirect effect: −0.139 [−0.193, −0.090], *p* < 0.0001 [non-parametric bootstrap]), as well WC, HC, WHR, and BF% (Table 3). However, for Olink_GDF15, this pattern was replicated only for BF% (indirect effect: −0.100 [−0.195, −0.010], *p* = 0.038 [non-parametric bootstrap]), and not the other adiposity measures.

**Table 3:** Mediating effect of GDF15 measured by Olink and SomaScan on the association between smoking and adiposity in 3638 Chinese adults.

Path	Olink		SomaScan	
	Beta (95% CI)	*p*-value	Beta (95% CI)	*p*-value
**BMI, kg/m^2^**				
Indirect (mediating) effect^a^	−0.026 (−0.070, 0.014)	0.20	−0.139 (−0.193, −0.090)	<0.0001
Direct effect^b^	−0.877 (−1.199, −0.543)	<0.0001	−0.764 (−1.071, −0.440)	<0.0001
**Waist circumference, cm**				
Indirect (mediating) effect^a^	−0.034 (−0.143, 0.082)	0.53	−0.340 (−0.492, −0.216)	<0.0001
Direct effect^b^	−1.594 (−2.458, −0.699)	<0.0001	−1.288 (−2.177, −0.431)	<0.0001
**Hip circumference, cm**				
Indirect (mediating) effect^a^	−0.052 (−0.134, 0.028)	0.20	−0.212 (−0.321, −0.123)	<0.0001
Direct effect^b^	−1.154 (−1.785, −0.540)	<0.0001	−0.994 (−1.544, −0.465)	<0.0001
**Waist/hip ratio**				
Indirect (mediating) effect^a^	0.000 (−0.001, 0.001)	0.97	−0.002 (−0.003, −0.001)	<0.0001
Direct effect^b^	−0.006 (−0.013, 0.002)	0.060	−0.005 (−0.011, 0.002)	0.14
**Body fat percentage, %**				
Indirect (mediating) effect^a^	−0.100 (−0.195, −0.010)	0.038	−0.341 (−0.470, −0.227)	<0.0001
Direct effect^b^	−0.852 (−1.491, −0.222)	0.004	−0.610 (−1.222, −0.010)	0.044

Indirect and direct effects obtained from mediation analyses on regression models adjusted for age, age^2^, sex, study area, education, case/subcohort ascertainment, plate ID (Olink only), ambient temperature, temperature^2^, fasting time, alcohol, physical activity, hypertension status, and kidney disease status. Data available for 3634 individuals for body fat percentage.

Abbreviations: BMI = body mass index; GDF15 = growth/differentiation factor 15.

a Indirect effect of smoking on adiposity measure through increasing GDF15.

b Direct effect of smoking on adiposity measure given GDF15.

The GDF15/RET ratio from both platforms significantly mediated some of the association of smoking on BMI (SomaScan indirect effect: −0.227 [−0.328, −0.145], *p* < 0.0001 [non-parametric bootstrap]), and all other adiposity measures (Table 4). Notably, the proportion of the indirect effect compared to the direct effect was greater for the GDF15/RET ratio compared to GDF15 alone in both platforms. Similarly, the GDF15/GFRAL ratio mediated the association between smoking and adiposity measures, except for WHR in both panels (Olink: *p* = 0.082, SomaScan: *p* = 0.074 [non-parametric bootstrap]) and WC in Olink (*p* = 0.062 [non-parametric bootstrap]). The GDF15/GFRAL ratio showed stronger indirect effects than GDF15 alone in the Olink panel, but weaker effects in the SomaScan panel. After taking account of the low prevalence of smoking among women, there were no clear sex-differences in these associations (Supplementary Tables S6–S8).

**Table 4:** Mediating effect of GDF15/RET and GDF15/GFRAL ratios on the association between smoking and adiposity measures in 3638 Chinese adults.

Path	Olink		SomaScan	
	Beta (95% CI)	*p*-value	Beta (95% CI)	*p*-value
**GDF15/RET ratio**				
**BMI, kg/m^2^**				
Indirect (mediating) effect^a^	−0.118 (−0.184, −0.062)	<0.0001	−0.227 (−0.328, −0.145)	<0.0001
Direct effect^b^	−0.786 (−1.101, −0.456)	<0.0001	−0.786 (−0.985, −0.375)	<0.0001
**Waist circumference, cm**				
Indirect (mediating) effect^a^	−0.319 (−0.486, −0.159)	<0.0001	−0.593 (−0.856, −0.359)	<0.0001
Direct effect^b^	−1.309 (−2.221, −0.460)	<0.0001	−1.034 (−1.923, −0.231)	0.012
**Hip circumference, cm**				
Indirect (mediating) effect^a^	−0.158 (−0.252, −0.076)	<0.0001	−0.333 (−0.468, −0.209)	<0.0001
Direct effect^b^	−1.048 (−1.633, −0.447)	<0.0001	−0.874 (−1.454, −0.251)	0.002
**Waist/hip ratio**				
Indirect (mediating) effect^a^	−0.002 (−0.003, −0.001)	0.002	−0.003 (−0.005, −0.002)	<0.0001
Direct effect^b^	−0.004 (−0.011, 0.002)	0.18	−0.003 (−0.010, 0.004)	0.352
**Body fat percentage, %**				
Indirect (mediating) effect^a^	−0.269 (−0.416, −0.125)	<0.0001	−0.486 (−0.671, −0.299)	<0.0001
Direct effect^b^	−0.683 (−1.217, −0.087)	0.024	−0.466 (−1.029, 0.072)	0.090
**GDF15/GFRAL ratio**				
**BMI, kg/m^2^**				
Indirect (mediating) effect^a^	−0.054 (−0.098, −0.020)	0.004	−0.080 (−0.126, −0.044)	<0.0001
Direct effect^b^	−0.849 (−1.162, −0.546)	<0.0001	−0.968 (−1.134, −0.530)	<0.0001
**Waist circumference, cm**				
Indirect (mediating) effect^a^	−0.087 (−0.193, 0.008)	0.062	−0.173 (−0.296, −0.067)	0.004
Direct effect^b^	−1.540 (−2.418, −0.718)	<0.0001	−1.455 (−2.335, −0.600)	0.004
**Hip circumference, cm**				
Indirect (mediating) effect^a^	−0.173 (−0.264, −0.094)	<0.0001	−0.136 (−0.218, −0.064)	<0.0001
Direct effect^b^	−1.033 (−1.670, −0.424)	<0.0001	−1.071 (−1.673, −0.446)	<0.0001
**Waist/hip ratio**				
Indirect (mediating) effect^a^	0.001 (0.000, 0.001)	0.082	−0.001 (−0.001, 0.000)	0.074
Direct effect^b^	−0.007 (−0.013, −0.001)	0.028	−0.006 (−0.012, 0.001)	0.088
**Body fat percentage, %**				
Indirect (mediating) effect^a^	−0.124 (−0.206, −0.051)	<0.0001	−0.194 (−0.305, −0.105)	<0.0001
Direct effect^b^	−0.828 (−1.425, −0.263)	0.002	−0.758 (−1.388, −0.118)	0.008

Indirect and direct effects obtained from mediation analyses on regression models adjusted for age, age^2^, sex, study area, education, case/subcohort ascertainment, plate ID (Olink only), ambient temperature, temperature^2^, fasting time, alcohol, physical activity, hypertension status, and kidney disease status. Data available for 3634 individuals for body fat percentage.

Abbreviations: BMI = body mass index; GDF15 = growth/differentiation factor 15; GFRAL = GDNF family receptor alpha; RET = proto-oncogene tyrosine-protein kinase receptor Ret.

a Indirect effect of smoking on adiposity measure through increasing GDF15/RET or GDF15/GFRAL ratios.

b Direct effect of smoking on adiposity measure given GDF15/RET or GDF15/GFRAL ratios.

Results from the difference-in-coefficients methods followed the patterns observed in the main mediation analyses (Table 5). Inclusion of the GDF15/RET ratio resulted in the greatest attenuation of the association between smoking and adiposity measures and SomaScan_GDF15 consistently attenuated effect sizes more markedly compared to Olink_GDF15.

**Table 5:** Changes in strength of the association between current smoking and adiposity measures with stepwise adjustment for GDF15, GDF15/RET ratio and GDF15/GFRAL ratio in 3638 Chinese adults.

Measure of adiposity	Beta (95% CI)	*p*-value	% change
**BMI, kg/m^2^**			
Adjusted model^a^	−0.903 (−1.232, −0.574)	7.88 × 10^−8^	*Reference*
+ Olink_GDF15	−0.877 (−1.209, −0.546)	2.19 × 10^−7^	2.9
+ SomaScan_GDF15	−0.764 (−1.094, −0.435)	5.63 × 10^−6^	15.4
+ Olink_GDF15/RET ratio	−0.786 (−1.111, −0.460)	2.29 × 10^−6^	13.0
+ SomaScan_GDF15/RET ratio	−0.676 (−0.996, −0.357)	3.36 × 10^−5^	25.1
+ Olink_GDF15/GFRAL ratio	−0.849 (−1.180, −0.519)	4.94 × 10^−7^	6.0
+ SomaScan_GDF15/GFRAL ratio	−0.823 (−1.153, −0.492)	1.08 × 10^−6^	8.9
**Waist circumference, cm**			
Adjusted model^a^	−1.628 (−2.554, −0.701)	5.79 × 10^−4^	*Reference*
+ Olink_GDF15	−1.594 (−2.526, −0.661)	8.17 × 10^−4^	2.1
+ SomaScan_GDF15	−1.287 (−2.216, −0.358)	6.64 × 10^−3^	21.0
+ Olink_GDF15/RET ratio	−1.309 (−2.226, −0.392)	5.16 × 10^−3^	19.6
+ SomaScan_GDF15/RET ratio	−1.034 (−1.937, −0.131)	0.02	36.5
+ Olink_GDF15/GFRAL ratio	−1.540 (−2.472, −0.609)	1.19 × 10^−3^	5.4
+ SomaScan_GDF15/GFRAL ratio	−1.455 (−2.386, −0.525)	2.19 × 10^−3^	10.6
**Hip circumference, cm**			
Adjusted model^a^	−1.206 (−1.841, −0.571)	2.00 × 10^−4^	*Reference*
+ Olink_GDF15	−1.154 (−1.793, −5.145)	4.08 × 10^−4^	4.3
+ SomaScan_GDF15	−0.994 (−1.631, −0.357)	2.25 × 10^−3^	17.6
+ Olink_GDF15/RET ratio	−1.048 (−1.681, −0.416)	1.17 × 10^−3^	13.1
+ SomaScan_GDF15/RET ratio	−0.874 (−1.499, −2.480)	6.21 × 10^−3^	27.5
+ Olink_GDF15/GFRAL ratio	−1.033 (−1.669, −3.968)	1.47 × 10^−3^	14.3
+ SomaScan_GDF15/GFRAL ratio	−1.071 (−1.708, −0.433)	1.01 × 10^−3^	11.2
**Waist/hip ratio**			
Adjusted model^a^	−0.006 (−0.013, 0.000)	0.06	*Reference*
+ Olink_GDF15	−0.006 (−0.013, 0.000)	0.06	0.0
+ SomaScan_GDF15	−0.005 (−0.012, 0.002)	0.18	16.7
+ Olink_GDF15/RET ratio	−0.004 (−0.011, 0.002)	0.20	33.3
+ SomaScan_GDF15/RET ratio	−0.003 (−0.010, 0.004)	0.37	50.0
+ Olink_GDF15/GFRAL ratio	−0.007 (−0.014, 0.000)	0.04	16.7
+ SomaScan_GDF15/GFRAL ratio	−0.006 (−0.012, 0.001)	0.10	0.0
**Body fat percentage, %**			
Adjusted model^a^	−0.952 (−1.609, −0.296)	4.59 × 10^−3^	*Reference*
+ Olink_GDF15	−0.852 (−1.513, −0.192)	0.01	10.5
+ SomaScan_GDF15	−0.611 (−1.266, 0.044)	0.07	35.8
+ Olink_GDF15/RET ratio	−0.683 (−1.329, −0.037)	0.04	28.3
+ SomaScan_GDF15/RET ratio	−0.467 (−1.100, 0.166)	0.15	51.0
+ Olink_GDF15/GFRAL ratio	−0.828 (−1.487, −0.169)	0.01	13.0
+ SomaScan_GDF15/GFRAL ratio	−0.757 (−1.415, −0.099)	0.02	20.5

Former smokers (n = 298) were excluded. Data is available for 3934 individuals for body fat percentage.

Each + represents the respective protein separately added to the model, and subsequent attenuation of the beta and 95% CI.

Abbreviations: BMI = body mass index; GDF15 = growth/differentiation factor 15; GFRAL = GDNF receptor alpha-like, RET = proto-oncogene tyrosine-protein kinase receptor Ret.

a Linear regression model is adjusted for age (linear and squared terms), sex, study area, education, case/subcohort ascertainment, plate ID, ambient temperature (linear and squared terms), fasting time, alcohol, physical activity, hypertension status and kidney disease status.

## Discussion

In this study of approximately 4000 Chinese adults, current cigarette smoking was associated with lower adiposity measures while ex-smokers had the highest. Smoking was associated with higher plasma GDF15 levels and levels of ratios of GDF15 with its receptors, particularly among individuals who smoked more on the assessment day. Although no clear association was observed between smoking and GFRAL or RET, RET showed a positive association with higher adiposity measures. MR analyses provided support for the causal relevance of smoking intensity for lower BMI in East Asians, and elevated GDF15. Notably, the GDF15-adiposity association differed somewhat between Olink and SomaScan, despite their overall high correlations, suggesting possible platform-specific variations. Although GDF15 partly mediated the association of smoking with adiposity, the GDF15/RET ratio emerged as the most consistent marker linking smoking with adiposity, suggesting its potential functional relevance for mediating the metabolic effects of smoking beyond GDF15 alone.

Previous epidemiological studies have consistently reported an association between smoking and lower adiposity levels. For example, in the Scottish Health Survey (n = 9047), current smokers had a mean BMI 1.2 kg/m^2^ lower in males and 1.0 kg/m^2^ lower in females compared to never smokers, consistent with the 0.9 kg/m^2^ difference observed in this study of Chinese adults.^40^ In UKB, current smokers were less likely to be obese, supported by a separate UKB study providing genetic evidence of lower BMI in current smokers.^41^^,^^42^ In the present study the observed differences in the genetic associations of smoking intensity with adiposity between East Asians and Europeans may stem from the GSCAN exposure dataset not including never-smokers and including ex-smokers, both of whom tend to show higher adiposity than current smokers. Additionally, the higher proportion of ex-smokers among ever-smokers in Europeans compared to East Asians could contribute to these differences.^43^, ^44^, ^45^ Nevertheless, the present findings, particularly those from MR analyses, provide further strong evidence for a causal association between smoking and adiposity in Chinese adults.

Preclinical animal studies have linked GDF15 to lower body weight through suppression of food intake and slowed gastric emptying.^11^ Evidence in humans, however, remains limited with conflicting findings. Two randomised controlled trials (RCTs) reported modest weight loss following GDF15 analogue treatment, although the effects were notably less pronounced compared to GLP-1R agonists such as Semaglutide.^46^, ^47^, ^48^ In contrast, MR analysis found no evidence for causality between GDF15 levels and BMI.^49^ Conversely, an RCT targeting GDF15 inhibition for cancer cachexia treatment observed weight gain after 12 weeks, indicating a role for GDF15 in weight regulation.^50^ Additionally, clinical studies also suggested that GDF15 mediates metformin-induced appetite suppression and weight loss among patients with diabetes.^51^^,^^52^

In this study, the GDF15/RET ratio was consistently inversely associated with lower adiposity levels, with effect sizes greater than GDF15 alone. Given the observed inverse correlation between these proteins, the ratio may capture RET receptor internalisation into cells and activation of downstream signal transduction pathways that may provide a more precise indicator of GDF15's role in weight regulation.^53^^,^^54^ However, RET, a widely expressed protein, forms complexes with other GDNF family members beyond GFRAL, and the full range of factors influencing RET or GFRAL plasma levels remain unclear.^53^ Nevertheless, RET levels associated positively with adiposity, while other GDNF ligands have not been implicated in adiposity-related pathways to date.^55^^,^^56^

Concern about weight gain is one factor sustaining smoking habits in both European and Chinese populations.^57^, ^58^, ^59^ However, as observed in this study and previous research, the weight differences between smokers and non-smokers are modest and outweighed by the many adverse effects of tobacco use.^60^ Despite this, public health messaging on the dangers of smoking has had limited success in China,^61^ suggesting that pharmacological interventions may aid reductions in smoking rates through mitigating post-cessation weight gain. Agents such as metformin, that is low-cost with an assuring safety profile, that promote endogenous GDF15 expression could help mitigate post-cessation weight gain and encourage higher quitting rates. Such interventions could, in turn, significantly reduce the global smoking-related disease burden projected for the 21st century.

This present study has several strengths. First, the detailed smoking data, validated through COex measurements, enabled comprehensive exploration of the smoking-GDF15 association, suggesting higher GDF15 levels upon acute exposure, that were consistent across all age and BMI groups studied. Additionally, the inclusion of multiple adiposity measures facilitated the assessment of both general and central adiposity, which were broadly consistent throughout the study. Third, the combined use of one-sample and two-sample MR provided complementary strengths: the one-sample approach accounted for the genetic instrument being derived from smokers, while the two-sample MR reduced the risk of bias from effect size inflation inherent to one-sample settings. Finally, levels of GDF15, RET, GFRAL, and their ratios (GDF15/RET, GDF15/GFRAL) could be compared and their associations with smoking and adiposity cross-validated between the two proteomics platforms.

However, there are several limitations. First, generalisability may be limited due to differences in smoking behaviour and adiposity between East Asian and other, for example, Western populations.^62^ Second, the relatively small sample size and low number of female smokers may limit the robustness of some findings, though results in females were broadly consistent with males. Third, RET and GFRAL levels represent their soluble forms and may not reflect the number of functional receptors on the cell membrane, which could have attenuated some findings. Fourth, the genetic instrument for daily smoking intensity could not be evaluated for GDF15/receptor ratios, and was developed in a cohort of ever-regular smokers, without outcome datasets specific to smokers, potentially introducing bias. One-sample analyses in ever-regular smokers addressed some concerns, with consistent findings for BMI and lung cancer, but not for IHD. Fifth, sample overlap, driven by inclusion of BBJ (52.3%) and CKB (30.2% for adiposity measures, 2.6% for protein levels) in GSCAN may have inflated Type-1 error in the two-sample MR by biasing MR estimates towards the observational association. However, the expected impact of this on the presented findings would be minimal since the estimated adjusted Type-1 error rate rises only marginally to 5.1%, reflecting the strength of the genetic instrument used.^63^ Additionally, there was no sample overlap in analysis of Europeans, which demonstrated similar associations for GDF15. Sixth, the genetic score for smoking intensity showed a strong negative association with alcohol consumption, in contrast with the previously reported positive observational association in CKB and the wider literature, and should be further investigated in future studies.^64^ Seventh, despite extensive efforts, we can only provide full validation of the first assumption of MR (through calculation of F-statistics). Finally, unmeasured or residual confounding, inherent to observational data, may violate some of the causal assumptions in the mediation analysis, and as such, these findings should be interpreted with caution.

Overall, the findings of the present study suggest that GDF15 may play a mediating role in smoking-related weight changes, with the GDF15/RET ratio providing additional insights. Future research should examine the relevance of circulating levels of RET or GFRAL receptor proteins, given their expression in the brain, and for RET, other tissues, and validate the potential of GDF15/receptor ratios as proxies for GDF15 activity. Moreover, experimental animal studies are needed to assess whether sustained GDF15 levels following tobacco exposure could mitigate post-cessation weight gain, which could inform discovery of therapeutic targets to minimise cessation-induced weight gain and enhance smoking cessation efforts.

## Contributors

Alexander C. Tinworth (conceptualisation, methodology, formal analysis, visualisation, writing—original draft, writing—review & editing). Andri Iona (conceptualisation, methodology, validation, visualisation, writing—review and editing), Pang Yao (conceptualisation, methodology, validation, visualisation, writing—review and editing), Iona Y. Millwood (Writing—review & editing, project administration), Hannah Fry (data curation, project administration), Jonathan Clarke (data curation), Baihan Wang (data curation), Mohsen Mazidi (project administration), Christiana Kartsonaki (writing—review & editing, project administration), Robin G. Walters (project administration), Huaidong Du (writing—review & editing, project administration), Canqing Yu (project administration), Yiping Chen (project administration), Dianjianyi Sun (project administration), Ling Yang (project administration), Dan Valle Schmidt (data curation), Jun Lv (project administration), Daniel Avery (data curation), Liming Li (project administration), Derrick A. Bennett (writing—review & editing, project administration), Richard Peto (project administration), Robert Clarke (writing—review and editing, project administration), Fiona Bragg (conceptualisation, methodology, validation, visualisation, writing—review and editing), Zhengming Chen (conceptualisation, methodology, validation, visualisation, writing—review and editing, project administration). Alexander C. Tinworth, Andri Iona, Pang Yao, Fiona Bragg and Zhengming Chen accessed and verified the underlying data reported in the manuscript. All authors read and approved the manuscript. Members of the China Kadoorie Collaborative Group provided administrative, technical, or research support.

## Data sharing statement

In CKB, non-genetic data (e.g., baseline, resurveys, biomarkers, and disease endpoints) are released periodically to *bona fide* researchers. Details of the CKB Data Sharing Policy, data release schedules and formal application procedures for accessing data are available at www.ckbbiobank.org. Accessing to individual participant genetic data (e.g., genotyping) is currently constrained by the Administrative Regulations on Human Genetic Resources of the People's Republic of China, which is usually through collaboration with CKB researchers. Summary statistics used in this study are publicly available from the following websites; the CKB PheWeb browser at: pheweb.ckbiobank.org; GSCAN: conservancy. umn.edu/items/91f6a003-6af2-4809-9785-53dc579dc788; the BioBank Japan PheWeb browser at: pheweb. jp; GIANT: portals. broadinstitute.org/collaboration/giant/index.php/GIANT_consortium; and the UKB: pan.ukbb.broadinstitute.org.

## Declaration of interests

The authors all declare no conflicts of interest.
