# Supplementary material for: GDF15 and its receptors as pathways mediating smoking related weight change
Source: eBioMedicine. 2025 Jun 16;117:105806. doi: 10.1016/j.ebiom.2025.105806 (PMC12209944; doi:10.1016/j.ebiom.2025.105806)
Supplement: Supplementary Figures and Tables [file mmc1.docx]

Supplementary Material

**GDF15 and its receptors as pathways mediating smoking related weight change**

**Contents**

[Members of the China Kadoorie Biobank Collaborative Group 3](#_Toc191649244)

[Supplementary Table 1: Genetic variants used to proxy daily smoking intensity in East Asians 4](#_Toc191649245)

[Supplementary Table 2: Genetic variants used to proxy daily smoking intensity in Europeans 5](#_Toc191649246)

[Supplementary Table 3: Associations of genetically-proxied daily smoking intensity with IHD, lung cancer, measures of adiposity and plasma levels of GDF15, RET and GFRAL in 2SMR analyses in East Asians 6](#_Toc191649247)

[Supplementary Table 4: Associations of genetically-predicted daily smoking intensity with IHD, lung cancer, measures of adiposity and plasma levels of GDF15, RET and GFRAL in 2SMR analyses in Europeans 8](#_Toc191649248)

[Supplementary Table 5: One-sample Mendelian randomization estimates for genetically-proxied daily smoking intensity and associations with adiposity and positive and negative controls in 34,603 ever-regular smokers in CKB 10](#_Toc191649249)

[Supplementary Table 6: Mediating effect of GDF15 on the association between smoking and adiposity in 3638 Chinese adults stratified by sex 11](#_Toc191649250)

[Supplementary Table 7: Mediating effect of the GDF15/RET ratio on the association between smoking and adiposity in 3638 Chinese adults stratified by sex 12](#_Toc191649251)

[Supplementary Table 8: Mediating effect of the GDF15/GFRAL ratio on the association between smoking and measures of adiposity in 3638 Chinese adults stratified by sex 13](#_Toc191649252)

[Supplementary Figure 1. Correlation between proteins across Olink and SomaScan platforms in 3936 Chinese adults 14](#_Toc191649253)

[Supplementary Figure 2. Associations of age with plasma levels of GDF15, RET, GFRAL and their ratios measured by Olink and SomaScan by smoking status in 1555 male Chinese adults 15](#_Toc191649254)

[Supplementary Figure 3. Associations of age with plasma levels of GDF15, RET, GFRAL and their ratios measured by Olink and SomaScan by smoking status in 2083 female Chinese adults 16](#_Toc191649255)

[Supplementary Figure 4. Associations of plasma levels of GDF15, RET, GFRAL and their ratios measured by Olink and SomaScan with BMI, by smoking status in 1555 Chinese males 17](#_Toc191649256)

[Supplementary Figure 5. Associations of plasma levels of GDF15, RET, GFRAL and their ratios measured by Olink and SomaScan with BMI, by smoking status in 2083 Chinese females 18](#_Toc191649257)

[Supplementary Figure 6. Associations of plasma levels of GDF15, RET and GFRAL and their ratios measured by Olink and Somascan, with WC by smoking status in 3638 Chinese adults 19](#_Toc191649258)

[Supplementary Figure 7. Associations of plasma levels of GDF15, RET, GFRAL and their ratios measured by Olink and SomaScan with HC, by smoking status in 3638 Chinese adults 20](#_Toc191649259)

[Supplementary Figure 8. Associations of plasma levels of GDF15, RET, GFRAL and their ratios measured by Olink and SomaScan with WHR, by smoking status in 3638 Chinese adults 21](#_Toc191649260)

[Supplementary Figure 9. Associations of plasma levels of GDF15, RET, GFRAL and their ratios measured by Olink and SomaScan with BF%, by smoking status in 3634 Chinese adults 22](#_Toc191649261)

[Supplementary Figure 10. Associations of smoking intensity with plasma levels of RET measured by Olink and SomaScan in 3638 Chinese adults 23](#_Toc191649262)

[Supplementary Figure 11. Associations of smoking intensity with plasma levels of the GDF15/RET ratio measured by Olink and SomaScan in 3638 Chinese adults 24](#_Toc191649263)

[Supplementary Figure 12. Associations of smoking intensity with plasma levels of GFRAL measured by Olink and Somascan in 3638 Chinese adults 25](#_Toc191649264)

[Supplementary Figure 13. Associations of smoking intensity with plasma levels of the GDF15/GFRAL ratio measured by Olink and SomaScan in 3638 Chinese adults 26](#_Toc191649265)

[Supplementary Figure 14. Associations of years since smoking cessation and plasma levels of GDF15, GDF15/RET and GDF15/GFRAL measured by Olink and SomaScan in 1485 Chinese adults 27](#_Toc191649266)

# **Members of the China Kadoorie Biobank Collaborative Group**

**International Steering Committee:** Junshi Chen, Zhengming Chen (PI), Robert Clarke, Rory Collins, Liming Li (PI), Jun Lv, Richard Peto, Robin Walters.

**International Co-ordinating Centre, Oxford:** Daniel Avery, Maxim Barnard, Derrick Bennett, Ruth Boxall, Ka Hung Chan, Yiping Chen, Zhengming Chen, Charlotte Clarke, Jonathan Clarke, Robert Clarke, Huaidong Du, Ahmed Edris Mohamed, Hannah Fry, Simon Gilbert, Pek Kei Im, Andri Iona, Maria Kakkoura, Christiana Kartsonaki, Hubert Lam, Kuang Lin, James Liu, Mohsen Mazidi, Iona Millwood, Sam Morris, Qunhua Nie, Alfred Pozarickij, Maryam Rahmati, Paul Ryder, Dan Schmidt, Becky Stevens, Iain Turnbull, Robin Walters, Baihan Wang, Lin Wang, Neil Wright, Ling Yang, Xiaoming Yang, Pang Yao.

**National Co-ordinating Centre, Beijing:** Xiao Han, Can Hou, Qingmei Xia, Chao Liu, Jun Lv, Pei Pei, Dianjanyi Sun, Canqing Yu, Lang Pan

**10 Regional Co-ordinating Centres:**

Qingdao CDC: Zengchang Pang, Ruqin Gao, Shanpeng Li, Haiping Duan, Shaojie Wang, Yongmei Liu, Ranran Du, Yajing Zang, Liang Cheng, Xiaocao Tian, Hua Zhang, Yaoming Zhai, Feng Ning, Xiaohui Sun, Feifei Li. Licang CDC: Silu Lv, Junzheng Wang, Wei Hou. Heilongjiang Provincial CDC: Wei Sun, Shichun Yan, Xiaoming Cui. Nangang CDC: Chi Wang, Zhenyuan Wu,Yanjie Li, Quan Kang. Hainan Provincial CDC: Huiming Luo, Tingting Ou. Meilan CDC: Xiangyang Zheng, Zhendong Guo, Shukuan Wu, Yilei Li, Huimei Li. Jiangsu Provincial CDC: Ming Wu, Yonglin Zhou, Jinyi Zhou, Ran Tao, Jie Yang, Jian Su. Suzhou CDC: Fang Liu, Jun Zhang, Yihe Hu, Yan Lu, Liangcai Ma, Aiyu Tang, Shuo Zhang, Jianrong Jin, Jingchao Liu. Guangxi Provincial CDC: Mei Lin, Zhenzhen Lu. Liuzhou CDC: Lifang Zhou, Changping Xie, Jian Lan,Tingping Zhu,Yun Liu, Liuping Wei, Liyuan Zhou, Ningyu Chen, Yulu Qin, Sisi Wang. Sichuan Provincial CDC: Xianping Wu, Ningmei Zhang, Xiaofang Chen, Xiaoyu Chang. Pengzhou CDC: Mingqiang Yuan, Xia Wu, Xiaofang Chen, Wei Jiang, Jiaqiu Liu, Qiang Sun. Gansu Provincial CDC: Faqing Chen, Xiaolan Ren, Caixia Dong. Maiji CDC: Hui Zhang, Enke Mao, Xiaoping Wang, Tao Wang, Xi zhang. Henan Provincial CDC: Kai Kang, Shixian Feng, Huizi Tian, Lei Fan. Huixian CDC: XiaoLin Li, Huarong Sun, Pan He, Xukui Zhang. Zhejiang Provincial CDC: Min Yu, Ruying Hu, Hao Wang. Tongxiang CDC: Xiaoyi Zhang, Yuan Cao, Kaixu Xie, Lingli Chen, Dun Shen. Hunan Provincial CDC: Xiaojun Li, Donghui Jin, Li Yin, Huilin Liu, Zhongxi Fu. Liuyang CDC: Xin Xu, Hao Zhang, Jianwei Chen,Yuan Peng, Libo Zhang, Chan Qu.

# **Supplementary Table 1: Genetic variants used to proxy daily smoking intensity in East Asians**

| **RSID** | **Chr** | **Pos (38)** | **EA** | **OA** | **EAF** | **Beta** | **SE** | ***p*-value** | **F stat** |
| --- | --- | --- | --- | --- | --- | --- | --- | --- | --- |
| rs1051730 | 15 | 78601997 | A | G | 0.027 | 0.078 | 0.014 | 1.90x10^-8^ | 31.3 |
| rs11066015 | 12 | 111730205 | A | G | 0.174 | 0.037 | 0.005 | 7.33x10^-10^ | 54.4 |
| rs11066325 | 12 | 112492671 | C | T | 0.175 | 0.035 | 0.005 | 8.51x10^-12^ | 49.3 |
| rs116873087 | 12 | 112074109 | C | G | 0.175 | 0.036 | 0.005 | 6.04x10^-12^ | 50.5 |
| rs116921376 | 19 | 40827821 | G | C | 0.145 | -0.103 | 0.006 | 9.97x10^-69^ | 293.9 |
| rs117121174 | 12 | 110259525 | A | G | 0.075 | 0.043 | 0.007 | 3.52x10^-10^ | 37.7 |
| rs12227162 | 12 | 110929440 | T | C | 0.149 | 0.045 | 0.006 | 5.02x10^-16^ | 57.0 |
| rs12979898 | 19 | 41018227 | A | G | 0.305 | -0.028 | 0.005 | 6.93x10^-9^ | 32.3 |
| rs140019668 | 19 | 40976315 | A | G | 0.012 | -0.107 | 0.016 | 9.92x10^-12^ | 44.7 |
| rs141200525 | 19 | 40655689 | A | C | 0.051 | 0.078 | 0.010 | 1.22x10^-15^ | 61.3 |
| rs141777035 | 19 | 40824267 | A | G | 0.087 | 0.055 | 0.007 | 2.29x10^-13^ | 61.0 |
| rs144252600 | 19 | 40932725 | G | A | 0.043 | 0.105 | 0.012 | 3.85x10^-18^ | 76.5 |
| rs145429030 | 19 | 41047701 | C | T | 0.038 | -0.088 | 0.011 | 4.98x10^-16^ | 63.8 |
| rs149425442 | 19 | 40812882 | T | A | 0.012 | 0.146 | 0.021 | 3.20x10^-12^ | 48.3 |
| rs149477457 | 19 | 40810757 | T | C | 0.017 | 0.154 | 0.019 | 2.31x10^-16^ | 65.7 |
| rs185308415 | 19 | 40811274 | G | A | 0.102 | -0.060 | 0.007 | 4.36x10^-20^ | 73.9 |
| rs2431413 | 19 | 40844073 | G | A | 0.258 | -0.032 | 0.005 | 1.42x10^-10^ | 41.5 |
| rs2604913 | 19 | 40712706 | A | G | 0.705 | -0.042 | 0.005 | 3.81x10^-15^ | 69.2 |
| rs2644891 | 19 | 40828466 | C | T | 0.776 | -0.071 | 0.006 | 1.09x10^-34^ | 140.6 |
| rs4803402 | 19 | 40906280 | A | G | 0.740 | 0.085 | 0.005 | 6.05x10^-69^ | 288.2 |
| rs59449188 | 19 | 40686712 | C | T | 0.302 | -0.058 | 0.005 | 7.78x10^-37^ | 133.9 |
| rs66889044 | 19 | 40832807 | T | C | 0.075 | 0.086 | 0.009 | 3.05x10^-22^ | 92.1 |
| rs71358934 | 19 | 40818173 | A | G | 0.344 | 0.039 | 0.005 | 1.13x10^-17^ | 60.2 |
| rs75025271 | 19 | 41027276 | G | A | 0.051 | 0.046 | 0.007 | 8.67x10^-10^ | 43.0 |
| rs78277894 | 8 | 27571675 | A | G | 0.224 | 0.037 | 0.005 | 7.16x10^-15^ | 54.7 |
| rs78388975 | 19 | 40909913 | A | G | 0.034 | 0.095 | 0.011 | 2.15x10^-18^ | 74.2 |
| rs79105258 | 12 | 111280427 | A | C | 0.216 | 0.038 | 0.005 | 1.99x10^-14^ | 59.0 |
| rs79366653 | 19 | 40824257 | G | A | 0.016 | -0.099 | 0.017 | 2.20x10^-9^ | 34.0 |
| rs80004291 | 19 | 40618899 | C | G | 0.140 | -0.053 | 0.006 | 6.97x10^-20^ | 78.9 |
| rs8100418 | 19 | 40908458 | T | A | 0.304 | 0.093 | 0.005 | 9.41x10^-81^ | 344.9 |
| rs8192709 | 19 | 40991369 | T | C | 0.047 | -0.066 | 0.010 | 3.43x10^-11^ | 43.0 |
| rs925368 | 12 | 109953174 | C | T | 0.072 | 0.041 | 0.007 | 1.43x10^-8^ | 33.6 |
| rs938682 | 15 | 78604205 | A | G | 0.533 | 0.041 | 0.004 | 3.06x10^-21^ | 107.0 |

Abbreviations: Chr=chromosome; EA=effect allele; EAF=effect allele frequency; OA=other allele; Pos=position; SE=standard error.

# **Supplementary Table 2: Genetic variants used to proxy daily smoking intensity in Europeans**

| **RSID** | **Chr** | **Pos (38)** | **EA** | **OA** | **EAF** | **Beta** | **SE** | ***p*-value** | **F stat** |
| --- | --- | --- | --- | --- | --- | --- | --- | --- | --- |
| rs10106661 | 8 | 42998571 | A | G | 0.139 | -0.029 | 0.005 | 1.54x10^-8^ | 32.9 |
| rs1052035 | 15 | 78549011 | C | T | 0.054 | 0.062 | 0.008 | 8.44x10^-14^ | 60.6 |
| rs11076320 | 16 | 52063640 | A | C | 0.632 | -0.021 | 0.003 | 8.88x10^-10^ | 48.5 |
| rs112178027 | 17 | 29236995 | T | C | 0.182 | 0.026 | 0.004 | 1.52 x10^-8^ | 40.6 |
| rs112827102 | 15 | 78766626 | C | T | 0.097 | -0.054 | 0.008 | 5.22 x10^-13^ | 46.1 |
| rs11634042 | 15 | 78813008 | T | C | 0.424 | 0.045 | 0.003 | 6.17 x10^-41^ | 224.7 |
| rs117824460 | 19 | 40865575 | G | A | 0.034 | -0.108 | 0.011 | 1.51 x10^-22^ | 96.3 |
| rs12438734 | 15 | 78759793 | C | T | 0.085 | -0.039 | 0.006 | 4.18 x10^-12^ | 43.1 |
| rs12443170 | 15 | 78615394 | A | G | 0.125 | -0.071 | 0.005 | 9.00 x10^-49^ | 203.1 |
| rs13254578 | 8 | 42690703 | C | G | 0.777 | 0.035 | 0.004 | 1.07 x10^-18^ | 74.8 |
| rs143342381 | 8 | 42669629 | T | C | 0.027 | -0.061 | 0.011 | 7.16 x10^-9^ | 31.0 |
| rs146722791 | 15 | 78847849 | T | C | 0.073 | 0.049 | 0.006 | 1.69 x10^-15^ | 66.1 |
| rs147760547 | 15 | 78523788 | G | A | 0.024 | 0.082 | 0.011 | 1.47 x10^-14^ | 54.9 |
| rs1552220 | 19 | 41019750 | A | G | 0.275 | -0.024 | 0.004 | 7.49 x10^-11^ | 36.0 |
| rs1565735 | 8 | 27568560 | A | T | 0.200 | 0.023 | 0.004 | 4.33 x10^-8^ | 32.5 |
| rs189302121 | 15 | 78600079 | T | A | 0.033 | 0.070 | 0.009 | 2.41 x10^-16^ | 60.5 |
| rs2036527 | 15 | 78559273 | A | G | 0.374 | 0.096 | 0.003 | 2.21 x10^-166^ | 1014.1 |
| rs2229961 | 15 | 78588410 | A | G | 0.013 | 0.144 | 0.016 | 3.26 x10^-18^ | 81.0 |
| rs2273500 | 20 | 63355597 | C | T | 0.146 | 0.037 | 0.005 | 9.06 x10^-16^ | 55.9 |
| rs2519764 | 9 | 133628060 | A | C | 0.713 | -0.022 | 0.004 | 2.41 x10^-9^ | 28.9 |
| rs2561534 | 19 | 40702223 | C | T | 0.188 | -0.024 | 0.004 | 3.77 x10^-8^ | 34.5 |
| rs2644899 | 19 | 40797044 | T | G | 0.705 | 0.021 | 0.004 | 2.39 x10^-8^ | 26.8 |
| rs28399462 | 19 | 40844710 | A | G | 0.022 | -0.105 | 0.010 | 1.45 x10^-27^ | 110.2 |
| rs2869876 | 15 | 78772018 | A | C | 0.184 | -0.026 | 0.004 | 8.99 x10^-10^ | 42.2 |
| rs3025383 | 9 | 133637247 | C | T | 0.181 | -0.034 | 0.004 | 4.15 x10^-16^ | 73.5 |
| rs41280048 | 15 | 78549249 | A | G | 0.020 | -0.067 | 0.012 | 1.24 x10^-8^ | 30.9 |
| rs42197 | 16 | 52387756 | C | T | 0.724 | 0.021 | 0.004 | 7.18 x10^-9^ | 28.6 |
| rs4274224 | 11 | 113448730 | A | G | 0.546 | 0.019 | 0.003 | 2.68 x10^-8^ | 38.0 |
| rs4522419 | 16 | 89786899 | C | T | 0.603 | -0.021 | 0.003 | 6.91 x10^-10^ | 49.0 |
| rs4911543 | 20 | 32107342 | G | T | 0.372 | 0.019 | 0.003 | 6.44 x10^-9^ | 41.8 |
| rs59586387 | 19 | 40869125 | G | C | 0.074 | -0.042 | 0.007 | 3.34 x10^-9^ | 36.2 |
| rs6141293 | 20 | 32361562 | C | T | 0.351 | 0.023 | 0.003 | 4.95 x10^-12^ | 60.3 |
| rs61884324 | 11 | 46741267 | A | G | 0.097 | 0.034 | 0.006 | 2.59 x10^-9^ | 31.4 |
| rs62010961 | 15 | 78814506 | G | C | 0.123 | -0.032 | 0.005 | 9.57 x10^-10^ | 39.7 |
| rs72740960 | 15 | 78559382 | G | A | 0.022 | 0.078 | 0.011 | 4.01 x10^-12^ | 50.5 |
| rs72745113 | 15 | 78716624 | A | G | 0.040 | 0.060 | 0.009 | 2.87 x10^-10^ | 43.7 |
| rs7507400 | 19 | 40824274 | T | G | 0.319 | -0.020 | 0.004 | 1.87 x10^-8^ | 26.0 |
| rs76474922 | 15 | 78592211 | C | A | 0.096 | -0.086 | 0.006 | 1.73 x10^-43^ | 207.1 |
| rs7872903 | 9 | 133619170 | C | T | 0.213 | 0.028 | 0.004 | 7.87 x10^-13^ | 49.7 |
| rs78963847 | 15 | 78421830 | C | G | 0.023 | 0.070 | 0.012 | 5.02 x10^-9^ | 33.5 |
| rs79409323 | 15 | 78872294 | C | T | 0.023 | -0.064 | 0.009 | 1.93 x10^-11^ | 49.9 |
| rs8039661 | 15 | 78966667 | T | C | 0.399 | -0.018 | 0.003 | 4.52 x10^-8^ | 37.6 |

Abbreviations: Chr=chromosome; EA=effect allele; EAF=effect allele frequency; OA=other allele; Pos=position; SE=standard error.

# **Supplementary Table 3: Associations of genetically-proxied daily smoking intensity with IHD, lung cancer, measures of adiposity and plasma levels of GDF15, RET and GFRAL in 2SMR analyses in East Asians**

| **Outcome, method** | **Beta (95% CI)** | ***p*-value** | **Egger test (*p*-value)** | **No. of PRESSO outliers** | **Egger test after excluding PRESSO outliers** |
| --- | --- | --- | --- | --- | --- |
| **IHD** |  |  | 0.190 (0.01) | 19 | -0.03 (0.27) |
| IVW | 1.151 (0.206, 2.095) | 0.017 |  |  |  |
| Weighted median | 0.128 (-0.046, 0.302) | 0.16 |  |  |  |
| MR-Egger | -1.907 (1.088, -4.039) | 0.09 |  |  |  |
| MR-PRESSO | 0.510 (0.111, 0.292) | 6.03x10^-4^ |  |  |  |
| **Lung cancer** |  |  | 0.03 (0.07) | 2 | NA |
| IVW | 1.600 (1.347, 1.761) | 2.19x10^-35^ |  |  |  |
| Weighted median | 1.430 (1.099, 1.761) | 2.23x10^-9^ |  |  |  |
| MR-Egger | 1.050 (0.435, 1.665) | 0.002 |  |  |  |
| MR-PRESSO | 1.620 (1.422, 1.818) | 1.36x10^-15^ |  |  |  |
| **BMI** |  |  | -0.001 (0.02) | 5 | -0.01 (0.13) |
| IVW | -0.062 (-0.109, -0.016) | 0.009 |  |  |  |
| Weighted median | -0.052 (-0.099, -0.006) | 0.040 |  |  |  |
| MR-Egger | 0.062 (-0.048, 0.173) | 0.28 |  |  |  |
| MR-PRESSO | -0.044 (-0.078, -0.010) | 0.017 |  |  |  |
| **WC** |  |  | -0.024 (0.01) | 8 | NA |
| IVW | -0.131 (-0.260, -0.002) | 0.048 |  |  |  |
| Weighted median | -0.006 (-0.065, 0.053) | 0.85 |  |  |  |
| MR-Egger | 0.248 (-0.056, 0.552) | 0.12 |  |  |  |
| MR-PRESSO | -0.015 (-0.056, 0.026) | 0.49 |  |  |  |
| **HC** |  |  | -0.018 (0.01) | 6 | NA |
| IVW | -0.096 (-0.192, 0.000) | 0.050 |  |  |  |
| Weighted median | -0.012 (-0.075, 0.051) | 0.70 |  |  |  |
| MR-Egger | 0.195 (-0.028, 0.418) | 0.10 |  |  |  |
| MR-PRESSO | -0.029 (-0.076, 0.018) | 0.25 |  |  |  |
| **WHR** |  |  | -0.021 (0.02) | 9 | NA |
| IVW | -0.117 (-0.239, 0.005) | 0.060 |  |  |  |
| Weighted median | -0.003 (-0.062, 0.056) | 0.92 |  |  |  |
| MR-Egger | 0.216 (-0.074, 0.506) | 0.15 |  |  |  |
| MR-PRESSO | -0.035 (-0.084, 0.014) | 0.18 |  |  |  |
| **BF%** |  |  | -0.010 (0.10) | 3 | NA |
| IVW | -0.074 (-0.158, 0.010) | 0.080 |  |  |  |
| Weighted median | -0.017 (-0.080, 0.046) | 0.59 |  |  |  |
| MR-Egger | 0.091 (-0.117, 0.299) | 0.40 |  |  |  |
| MR-PRESSO | -0.032 (-0.089, 0.025) | 0.29 |  |  |  |

Estimates reflect a 1-category higher in category of cigarettes smoked per day, with 1-5 cigarettes as the baseline. For IHD and lung cancer, the beta represents the log odds ratio per category higher in genetically proxied daily smoking intensity. For continuous outcomes, the beta represents the standard deviation change per category higher. Significant associations with evidence of horizontal pleiotropy (Egger test *p*<0.05) that remained significant after MR-PRESSO adjustments were re-evaluated by repeating the Egger test after excluding MR-PRESSO identified outlier variants.

BF%=body fat percentage; BMI=body mass index; GDF15=growth/differentiation factor 15; GFRAL=GDNF family receptor alpha-like; HC=hip circumference; IHD=ischaemic heart disease; RET=protein-oncogene tyrosine kinase receptor Ret; WC=waist circumference; WHR=waist/hip ratio.

**Supplementary Table 3 continued**

| **Outcome, method** | **Beta (95% CI)** | ***p*-value** | **Egger test (*p*-value)** | **No. of PRESSO outliers** | **Egger test after excluding PRESSO outliers** |
| --- | --- | --- | --- | --- | --- |
| **Olink proteins** |  |  |  |  |  |
| **GDF15** |  |  | 0.007 (0.64) | 0 | NA |
| IVW | 0.245 (0.100, 0.390) | 9.23x10^-4^ |  |  |  |
| Weighted median | 0.321 (0.037, 0.605) | 0.027 |  |  |  |
| MR-Egger | 0.134 (-0.366, 0.634) | 0.60 |  |  |  |
| MR-PRESSO | 0.245 (0.100, 0.390) | 0.002 |  |  |  |
| **RET** |  |  | 0.03 (0.11) | 0 | NA |
| IVW | 0.365 (0.112, 0.618) | 0.005 |  |  |  |
| Weighted median | 0.337 (0.010, 0.664) | 0.043 |  |  |  |
| MR-Egger | -0.104 (-0.819, 0.511) | 0.74 |  |  |  |
| MR-PRESSO | 0.365 (0.112, 0.618) | 0.008 |  |  |  |
| **GFRAL** |  |  | 0.04 (0.04) | 0 | NA |
| IVW | 0.041 (-0.228, 0.310) | 0.77 |  |  |  |
| Weighted median | -0.203 (-0.493, 0.087) | 0.17 |  |  |  |
| MR-Egger | -0.601 (-1.236, 0.034) | 0.07 |  |  |  |
| MR-PRESSO | 0.041 (-0.228, 0.310) | 0.77 |  |  |  |
| **SomaScan proteins** |  |  |  |  |  |
| **GDF15** |  |  | 0.04 (0.01) | 0 | NA |
| IVW | 0.354 (0.158, 0.550) | 4.10x10^-4^ |  |  |  |
| Weighted median | 0.246 (-0.038, 0.530) | 0.09 |  |  |  |
| MR-Egger | -0.282 (-0.772, 0.208) | 0.27 |  |  |  |
| MR-PRESSO | 0.354 (0.158, 0.550) | 0.001 |  |  |  |
| **RET** |  |  | 0.05 (0.04) | 1 | NA |
| IVW | 0.159 (-0.139, 0.457) | 0.30 |  |  |  |
| Weighted median | -0.168 (-0.470, 0.134) | 0.28 |  |  |  |
| MR-Egger | -0.555 (-1.263, 0.153) | 0.13 |  |  |  |
| MR-PRESSO | 0.111 (-0.173, 0.395) | 0.45 |  |  |  |
| **GFRAL** |  |  | 0.04 (0.02) | 0 | NA |
| IVW | 0.215 (-0.038, 0.468) | 0.09 |  |  |  |
| Weighted median | 0.042 (-0.234, 0.318) | 0.77 |  |  |  |
| MR-Egger | -0.441 (-1.031, 0.149) | 0.15 |  |  |  |
| MR-PRESSO | 0.215 (-0.038, 0.468) | 0.10 |  |  |  |

Estimates reflect a 1-category higher in category of cigarettes smoked per day, with 1-5 cigarettes as the baseline. For IHD and lung cancer, the beta represents the log odds ratio per category higher in genetically proxied daily smoking intensity. For continuous outcomes, the beta represents the standard deviation change per category higher. Significant associations with evidence of horizontal pleiotropy (Egger test *p*<0.05) that remained significant after MR-PRESSO adjustments were re-evaluated by repeating the Egger test after excluding MR-PRESSO identified outlier variants.

BF%=body fat percentage; BMI=body mass index; GDF15=growth/differentiation factor 15; GFRAL=GDNF family receptor alpha-like; HC=hip circumference; IHD=ischaemic heart disease; RET=protein-oncogene tyrosine kinase receptor Ret; WC=waist circumference; WHR=waist/hip ratio.

# **Supplementary Table 4: Associations of genetically-predicted daily smoking intensity with IHD, lung cancer, measures of adiposity and plasma levels of GDF15, RET and GFRAL in 2SMR analyses in Europeans**

| **Outcome, method** | **Beta (95% CI)** | ***p*-value** | **Egger test (*p*-value)** | **No. of PRESSO outliers** | **Egger test after excluding PRESSO outliers** |
| --- | --- | --- | --- | --- | --- |
| **IHD** |  |  | 0.008 (0.22) | 5 | NA |
| IVW | -0.153 (-0.335, 0.028) | 0.10 |  |  |  |
| Weighted median | -0.109 (-0.269, 0.052) | 0.19 |  |  |  |
| MR-Egger | -0.235 (-0.574, 0.104) | 0.18 |  |  |  |
| MR-PRESSO | -0.034 (-0.167, 0.098) | 0.61 |  |  |  |
| **Lung cancer** |  |  |  |  | NA |
| IVW | 1.762 (0.695, 2.830) | 0.001 | 0.004 (0.93) | 0 |  |
| Weighted median | 0.988 (-0.592, 2.567) | 0.22 |  |  |  |
| MR-Egger | 1.683 (-0.318, 3.685) | 0.11 |  |  |  |
| MR-PRESSO | 1.762 (0.695, 2.830) | 0.002 |  |  |  |
| **BMI** |  |  |  |  | NA |
| IVW | -0.009 (-0.044, 0.026) | 0.62 | 0.002 (0.17) | 5 |  |
| Weighted median | -0.040 (-0.070, -0.010) | 0.010 |  |  |  |
| MR-Egger | -0.047 (-0.069, 0.017) | 0.16 |  |  |  |
| MR-PRESSO | 0.001 (-0.034, 0.037) | 0.94 |  |  |  |
| **WC** |  |  | 0.001 (0.21) | 0 | NA |
| IVW | 0.007 (-0.013, 0.027) | 0.65 |  |  |  |
| Weighted median | -0.026 (-0.058, 0.005) | 0.10 |  |  |  |
| MR-Egger | -0.022 (-0.075, 0.030) | 0.41 |  |  |  |
| MR-PRESSO | 0.001 (-0.026, 0.027) | 0.96 |  |  |  |
| **HC** |  |  | 0.002 (0.32) | 4 | NA |
| IVW | 0.004 (-0.037, 0.045) | 0.84 |  |  |  |
| Weighted median | 0.015 (-0.075, 0.022) | 0.29 |  |  |  |
| MR-Egger | -0.029 (-0.105, 0.048) | 0.47 |  |  |  |
| MR-PRESSO | 0.018 (-0.018, 0.054) | 0.34 |  |  |  |
| **WHR** |  |  | 0.001 (0.34) | 5 | NA |
| IVW | 0.010 (-0.007, 0.035) | 0.45 |  |  |  |
| Weighted median | 0.004 (-0.025, 0.033) | 0.79 |  |  |  |
| MR-Egger | -0.010 (-0.057, 0.037) | 0.68 |  |  |  |
| MR-PRESSO | 0.001 (-0.020, 0.023) | 0.90 |  |  |  |
| **BF%** |  |  | 0.001 (0.13) | 3 | NA |
| IVW | -0.004 (-0.029, 0.021) | 0.78 |  |  |  |
| Weighted median | -0.026 (-0.052, 0.000) | 0.053 |  |  |  |
| MR-Egger | -0.034 (-0.079, 0.012) | 0.16 |  |  |  |
| MR-PRESSO | -0.004 (-0.034, 0.007) | 0.22 |  |  |  |

Estimates reflect a 1-category higher in category of cigarettes smoked per day, with 1-5 cigarettes as the baseline. For IHD and lung cancer, the beta represents the log odds ratio per category higher in genetically proxied daily smoking intensity. For continuous outcomes, the beta represents the standard deviation change per category higher. Significant associations with evidence of horizontal pleiotropy (Egger test *p*<0.05) that remained significant after MR-PRESSO adjustments were re-evaluated by repeating the Egger test after excluding MR-PRESSO identified outlier variants.

BF%=body fat percentage; BMI=body mass index; GDF15=growth/differentiation factor 15; GFRAL=GDNF family receptor alpha-like; HC=hip circumference; IHD=ischaemic heart disease; RET=protein-oncogene tyrosine kinase receptor Ret; WC=waist circumference; WHR=waist/hip ratio.

**Supplementary Table 4 continued**

| **Outcome, method** | **Beta (95% CI)** | ***p*-value** | **Egger test (*p*-value)** | **No. of PRESSO outliers** | **Egger test after excluding PRESSO outliers** |
| --- | --- | --- | --- | --- | --- |
| **Olink proteins** |  |  |  |  |  |
| **GDF15** |  |  | 0.007 (0.02) | 0 | NA |
| IVW | 0.143 (0.078, 0.210) | 7.60x10^-4^ |  |  |  |
| Weighted median | 0.070 (-0.038, 0.177) | 0.20 |  |  |  |
| MR-Egger | -0.007 (-0.153, 0.138) | 0.92 |  |  |  |
| MR-PRESSO | 0.143 (0.078, 0.210) | 0.002 |  |  |  |
| **RET** |  |  | -0.006 (0.05) | 0 | NA |
| IVW | 0.048 (-0.024, 0.119) | 0.19 |  |  |  |
| Weighted median | 0.131 (0.025, 0.239) | 0.016 |  |  |  |
| MR-Egger | 0.168 (0.022, 0.305) | 0.021 |  |  |  |
| MR-PRESSO | 0.048 (-0.024, 0.119) | 0.20 |  |  |  |
| **GFRAL** |  |  | -0.004 (0.25) | 1 | NA |
| IVW | -0.035 (-0.125, 0.056) | 0.45 |  |  |  |
| Weighted median | -0.004 (-0.121, 0.113) | 0.95 |  |  |  |
| MR-Egger | 0.048 (-0.118, 0.215) | 0.57 |  |  |  |
| MR-PRESSO | -0.015 (-0.094, 0.064) | 0.71 |  |  |  |
| **Generation Scotland** |  |  |  |  |  |
| **GDF15** |  |  | -0.005 (0.27) | 0 | NA |
| IVW | 0.205 (0.090, 0.320) | 4.57x10^-4^ |  |  |  |
| Weighted median | 0.198 (0.034, 0.361) | 0.018 |  |  |  |
| MR-Egger | 0.306 (0.097, 0.515) | 0.007 |  |  |  |
| MR-PRESSO | 0. 205 (0.090, 0.320) | 0.001 |  |  |  |

Estimates reflect a 1-category higher in category of cigarettes smoked per day, with 1-5 cigarettes as the baseline. For IHD and lung cancer, the beta represents the log odds ratio per category higher in genetically proxied daily smoking intensity. For continuous outcomes, the beta represents the standard deviation change per category higher. Significant associations with evidence of horizontal pleiotropy (Egger test *p*<0.05) that remained significant after MR-PRESSO adjustments were re-evaluated by repeating the Egger test after excluding MR-PRESSO identified outlier variants.

BF%=body fat percentage; BMI=body mass index; GDF15=growth/differentiation factor 15; GFRAL=GDNF family receptor alpha-like; HC=hip circumference; IHD=ischaemic heart disease; RET=protein-oncogene tyrosine kinase receptor Ret; WC=waist circumference; WHR=waist/hip ratio.

# **Supplementary Table 5: One-sample Mendelian randomization estimates for genetically-proxied daily smoking intensity and associations with adiposity and positive and negative controls in 34,603 ever-regular smokers in CKB**

| **Outcome** | **Beta** | **Standard error** | ***p-*value** | **No. of participants or cases/total participants** |
| --- | --- | --- | --- | --- |
| Cigarettes per day | 2.652 | 0.249 | 1.99x10^-26^ | 34,603 |
| Ischaemic heart disease | 0.034 | 0.073 | 0.64 | 6348/27,467 |
| Lung cancer | 0.413 | 0.146 | 0.005 | 1002/25,032 |
| Body mass index, kg/m^2^ | -0.355 | 0.076 | 2.65x10^-6^ | 34,603 |
| Waist circumference, cm | -1.633 | 0.224 | 3.05x10^-13^ | 34,603 |
| Hip circumference, cm | -0.633 | 0.144 | 1.05x10^-5^ | 34,603 |
| Waist/hip ratio | -0.012 | 0.002 | 1.99x10^-14^ | 34,603 |
| Body fat percentage, % | -0.885 | 0.146 | 1.21x10^-9^ | 34,603 |
| Age | -0.275 | 0.251 | 0.27 | 34,603 |
| Female sex | 0.002 | 0.005 | 0.70 | 34,603 |
| Study area | 0.222 | 0.379 | 0.56 | 34,603 |
| Education | -0.007 | 0.021 | 0.94 | 34,603 |
| Physical activity, MET-h/day | 0.236 | 0.311 | 0.45 | 34,603 |
| Alcohol consumed per week, g | -133.6 | 4.763 | 3.31x10^-171^ | 34,603 |

Estimates reflect logistic or linear regression models between the weighted genetic score and positive and negative control outcomes adjusted where appropriate for age (linear and squared terms), sex, study area (10 groups), 11 genetic principal components, GWAS array and population subset status (continuous outcomes only).

| **Supplementary Table 6: Mediating effect of GDF15 on the association between smoking and adiposity in 3638 Chinese adults stratified by sex** | | | | | |
| --- | --- | --- | --- | --- | --- |
|  | **Olink** | |  | **SomaScan** | |
| **Path** | **Beta (95% CI)** | ***p*-value** |  | **Beta (95% CI)** | ***p*-value** |
| **Males (n=1555)** |  |  |  |  |  |
| **BMI, kg/m²** |  |  |  |  |  |
| Indirect (mediating) effect^a^ | -0.082 (-0.161, -0.013) | 0.008 |  | -0.199 (-0.285, -0.126) | <0.0001 |
| Direct effect^b^ | -0.668 (-0.668, -0.286) | <0.0001 |  | -0.553 (-1.925, -0.192) | 0.002 |
| **Waist circumference, cm** |  |  |  |  |  |
| Indirect (mediating) effect^a^ | -0.212 (-0.419, -0.024) | 0.032 |  | -0.526 (-0.773, -0.311) | <0.0001 |
| Direct effect^b^ | -0.966 (-2.054, 0.046) | 0.066 |  | -0.651 (-1.597, 0.341) | 0.186 |
| **Hip circumference, cm** |  |  |  |  |  |
| Indirect (mediating) effect^a^ | -0.087 (-0.228, 0.050) | 0.22 |  | -0.268 (-0.425, -0.135) | <0.0001 |
| Direct effect^b^ | -0.716 (-1.414, -0.030) | 0.046 |  | -0.532 (-1.253, 0.187) | 0.142 |
| **Waist/hip ratio** |  |  |  |  |  |
| Indirect (mediating) effect^a^ | -0.002 (-0.003, 0.000) | 0.016 |  | -0.003 (-0.005, -0.002) | <0.0001 |
| Direct effect^b^ | -0.004 (-0.011, 0.003) | 0.27 |  | -0.002 (-0.009, 0.005) | 0.52 |
| **Body fat percentage, %** |  |  |  |  |  |
| Indirect (mediating) effect^a^ | -0.181 (-0.322, -0.058) | 0.006 |  | -0.385 (-0.547, -0.235) | <0.0001 |
| Direct effect^b^ | -0.410 (-1.088, 0.234) | 0.21 |  | -0.207 (-0.799, 0.410) | 0.50 |
|  |  |  |  |  |  |
| **Females (n=2083)** |  |  |  |  |  |
| **BMI, kg/m²** |  |  |  |  |  |
| Indirect (mediating) effect^a^ | 0.000 (-0.040, 0.038) | 0.99 |  | -0.062 (-0.151, -0.002) | 0.040 |
| Direct effect^b^ | -1.250 (-1.992, -0.472) | <0.0001 |  | -1.180, (-1.929, -0.384) | 0.002 |
| **Waist circumference, cm** |  |  |  |  |  |
| Indirect (mediating) effect^a^ | 0.026 (-0.052, 0.150) | 0.55 |  | -0.125 (-0.326, -0.002) | 0.044 |
| Direct effect^b^ | -2.078 (-3.968, -0.206) | 0.036 |  | -1.949 (-3.951, 0.104) | 0.066 |
| **Hip circumference, cm** |  |  |  |  |  |
| Indirect (mediating) effect^a^ | -0.017 (-0.098, 0.042) | 0.57 |  | -0.104 (-0.259, -0.003) | 0.044 |
| Direct effect^b^ | -2.056 (-3.419, -0.646) | 0.008 |  | -1.963 (-3.382, -0.637) | 0.002 |
| **Waist/hip ratio** |  |  |  |  |  |
| Indirect (mediating) effect^a^ | 0.000 (0.000, 0.002) | 0.31 |  | 0.000 (-0.001, 0.000) | 0.27 |
| Direct effect^b^ | -0.002 (-0.017, 0.012) | 0.68 |  | -0.002 (-0.017, 0.013) | 0.81 |
| **Body fat percentage, %** |  |  |  |  |  |
| Indirect (mediating) effect^a^ | -0.036 (-0.154, 0.035) | 0.41 |  | -0.200 (-0.439, -0.017) | 0.030 |
| Direct effect^b^ | -2.276 (-3.876, -0.706) | 0.004 |  | -2.091 (-3.595, -0.593) | 0.004 |
| Indirect and direct effects obtained from mediation analyses on regression models adjusted for age, age², sex, study area, education, case/subcohort ascertainment, plate ID (Olink only), ambient temperature, temperature², fasting time, alcohol, physical activity, hypertension status, and kidney disease status. Data available for 3634 individuals for body fat percentage. ^a^Indirect effect of smoking on adiposity measure through increasing GDF15. ^b^Direct effect of smoking on adiposity measure given GDF15.  Abbreviations: BMI=body mass index; GDF15=growth/differentiation factor 15. | | | | | |

| **Supplementary Table 7: Mediating effect of the GDF15/RET ratio on the association between smoking and adiposity in 3638 Chinese adults stratified by sex** | | | | | |
| --- | --- | --- | --- | --- | --- |
|  | **Olink** | |  | **SomaScan** | |
| **Path** | **Beta (95% CI)** | ***p*-value** |  | **Beta (95% CI)** | ***p*-value** |
| **Males (n=1555)** |  |  |  |  |  |
| **BMI, kg/m²** |  |  |  |  |  |
| Indirect (mediating) effect^a^ | -0.111 (-0.191, -0.041) | <0.0001 |  | -0.211 (-0.318, -0.121) | <0.0001 |
| Direct effect^b^ | -0.640 (-0.984, -0.311) | <0.0001 |  | -0.542 (-0.853, -0.197) | 0.004 |
| **Waist circumference, cm** |  |  |  |  |  |
| Indirect (mediating) effect^a^ | -0.291 (-0.503, -0.117) | <0.0001 |  | -0.529 (-0.786, -0.297) | <0.0001 |
| Direct effect^b^ | -0.887 (-1.932, 0.102) | 0.074 |  | -0.648 (-1.582, 0.268) | 0.20 |
| **Hip circumference, cm** |  |  |  |  |  |
| Indirect (mediating) effect^a^ | -0.126 (-0.239, -0.037) | 0.002 |  | -0.273 (-0.415, -0.146) | <0.0001 |
| Direct effect^b^ | -0.677 (-1.363, 0.025) | 0.064 |  | -0.527 (-1.250, 0.126) | 0.14 |
| **Waist/hip ratio** |  |  |  |  |  |
| Indirect (mediating) effect^a^ | -0.002 (-0.003, -0.001) | <0.0001 |  | -0.003 (-0.005, -0.002) | <0.0001 |
| Direct effect^b^ | -0.004 (-0.011, 0.004) | 0.28 |  | -0.003 (-0.009, 0.005) | 0.48 |
| **Body fat percentage, %** |  |  |  |  |  |
| Indirect (mediating) effect^a^ | -0.226 (-0.400, -0.100) | <0.0001 |  | -0.388 (-0.585, -0.231) | <0.0001 |
| Direct effect^b^ | -0.365 (-1.014, 0.257) | 0.28 |  | -0.205 (-0.844, 0.394) | 0.54 |
|  |  |  |  |  |  |
| **Females (n=2083)** |  |  |  |  |  |
| **BMI, kg/m²** |  |  |  |  |  |
| Indirect (mediating) effect^a^ | -0.131 (-0.265, -0.006) | 0.042 |  | -0.165 (-0.355, 0.055) | 0.14 |
| Direct effect^b^ | -1.120 (-1.842, -0.389) | 0.004 |  | -1.078 (-1.775, -0.358) | 0.004 |
| **Waist circumference, cm** |  |  |  |  |  |
| Indirect (mediating) effect^a^ | -0.344 (-0.697, 0.026) | 0.072 |  | -0.424 (-0.974, 0.093) | 0.13 |
| Direct effect^b^ | -1.708 (-3.728, 0.387) | 0.096 |  | -1.650 (-3.790, 0.367) | 0.10 |
| **Hip circumference, cm** |  |  |  |  |  |
| Indirect (mediating) effect^a^ | -0.188 (-0.402, -0.013) | 0.032 |  | -0.256 (-0.577, 0.066) | 0.13 |
| Direct effect^b^ | -1.885 (-3.221, -0.494) | 0.012 |  | -1.812 (-3.138, -0.375) | 0.008 |
| **Waist/hip ratio** |  |  |  |  |  |
| Indirect (mediating) effect^a^ | -0.002 (-0.004, 0.000) | 0.034 |  | -0.002 (-0.005, 0.001) | 0.11 |
| Direct effect^b^ | 0.000 (-0.015, 0.015) | 0.97 |  | 0.000 (-0.015, 0.014) | 0.98 |
| **Body fat percentage, %** |  |  |  |  |  |
| Indirect (mediating) effect^a^ | -0.322 (-0.656, -0.009) | 0.046 |  | -0.381 (-0.907, 0.098) | 0.11 |
| Direct effect^b^ | -1.990 (-3.638, -0.397) | 0.020 |  | -1.911 (-3.448, -0.318) | 0.020 |
| Indirect and direct effects obtained from mediation analyses on regression models adjusted for age, age², sex, study area, education, case/subcohort ascertainment, plate ID (Olink only), ambient temperature, temperature², fasting time, alcohol, physical activity, hypertension status, and kidney disease status. Data available for 3634 individuals for body fat percentage. ^a^Indirect effect of smoking on adiposity measure through increasing GDF15/RET ratio. ^b^Direct effect of smoking on adiposity measure given GDF15/RET ratio.  Abbreviations: BMI=body mass index; GDF15=growth/differentiation factor 15; RET=proto-oncogene tyrosine-protein kinase receptor Ret. | | | | | |

| **Supplementary Table 8: Mediating effect of the GDF15/GFRAL ratio on the association between smoking and measures of adiposity in 3638 Chinese adults stratified by sex** | | | | | |
| --- | --- | --- | --- | --- | --- |
|  | **Olink** | |  | **SomaScan** | |
| **Path** | **Beta (95% CI)** | ***p*-value** |  | **Beta (95% CI)** | ***p*-value** |
| **Males (n=1555)** |  |  |  |  |  |
| **BMI, kg/m²** |  |  |  |  |  |
| Indirect (mediating) effect^a^ | -0.046 (-0.104, 0.000) | 0.054 |  | -0.102 (-0.170, -0.046) | <0.0001 |
| Direct effect^b^ | -0.705 (-1.046, -0.338) | <0.0001 |  | -0.650 (-1.007, -0.285) | <0.0001 |
| **Waist circumference, cm** |  |  |  |  |  |
| Indirect (mediating) effect^a^ | -0.114 (-0.278, 0.029) | 0.12 |  | -0.268 (-0.457, -0.120) | 0.002 |
| Direct effect^b^ | -1.063 (-2.061, -0.056) | 0.042 |  | -0.908 (-1.942, 0.019) | 0.054 |
| **Hip circumference, cm** |  |  |  |  |  |
| Indirect (mediating) effect^a^ | -0.118 (-0.243, -0.015) | 0.012 |  | -0.155 (-0.287, -0.042) | 0.008 |
| Direct effect^b^ | -0.685 (-1.457, 0.031) | 0.062 |  | -0.645 (-1.343, 0.044) | 0.066 |
| **Waist/hip ratio** |  |  |  |  |  |
| Indirect (mediating) effect^a^ | 0.000 (-0.001, 0.001) | 0.75 |  | -0.001 (-0.003, 0.000) | 0.004 |
| Direct effect^b^ | -0.006 (-0.012, 0.001) | 0.11 |  | -0.004 (-0.012, 0.003) | 0.23 |
| **Body fat percentage, %** |  |  |  |  |  |
| Indirect (mediating) effect^a^ | -0.099 (-0.213, 0.001) | 0.052 |  | -0.191 (-0.324, -0.080) | 0.002 |
| Direct effect^b^ | -0.491 (-1.114, 0.145) | 0.114 |  | -0.401 (-1.058, 0.227) | 0.25 |
|  |  |  |  |  |  |
| **Females (n=2083)** |  |  |  |  |  |
| **BMI, kg/m²** |  |  |  |  |  |
| Indirect (mediating) effect^a^ | -0.056 (-0.123, -0.005) | 0.022 |  | -0.061 (-0.130, -0.007) | 0.018 |
| Direct effect^b^ | -1.176 (-1.889, -0.410) | 0.006 |  | -1.181 (-1.903, -0.431) | <0.0001 |
| **Waist circumference, cm** |  |  |  |  |  |
| Indirect (mediating) effect^a^ | -0.057 (-0.229, 0.063) | 0.36 |  | -0.101 (-0.259, 0.029) | 0.11 |
| Direct effect^b^ | -1.955 (-4.014, 0.086) | 0.054 |  | -1.973 (-4.049, -0.196) | 0.074 |
| **Hip circumference, cm** |  |  |  |  |  |
| Indirect (mediating) effect^a^ | -0.198 (-0.387, -0.057) | <0.0001 |  | -0.107 (-0.234, -0.018) | 0.008 |
| Direct effect^b^ | -1.839 (-3.285, -0.416) | 0.006 |  | -1.960 (-3.319, -0.510) | 0.008 |
| **Waist/hip ratio** |  |  |  |  |  |
| Indirect (mediating) effect^a^ | 0.001 (0.000, 0.003) | 0.012 |  | 0.000 (-0.001, 0.001) | 0.74 |
| Direct effect^b^ | -0.003 (-0.018, 0.010) | 0.69 |  | -0.002 (-0.018, 0.013) | 0.74 |
| **Body fat percentage, %** |  |  |  |  |  |
| Indirect (mediating) effect^a^ | -0.125 (-0.264, -0.012) | 0.026 |  | -0.178 (-0.346, -0.059) | <0.0001 |
| Direct effect^b^ | -2.187 (-3.835, -0.689) | 0.006 |  | -2.114 (-3.722, -0.379) | 0.014 |
| Indirect and direct effects obtained from mediation analyses on regression models adjusted for age, age², sex, study area, education, case/subcohort ascertainment, plate ID (Olink only), ambient temperature, temperature², fasting time, alcohol, physical activity, hypertension status, and kidney disease status. Data available for 3634 individuals for body fat percentage. ^a^Indirect effect of smoking on adiposity measure through increasing GDF15/GFRAL ratio. ^b^Direct effect of smoking on adiposity measure given GDF15/GFRAL ratio.  Abbreviations: BMI=body mass index; GDF15=growth/differentiation factor 15; GFRAL=GDNF family receptor alpha-like. | | | | | |

# **Supplementary Figure 1. Correlation between proteins across Olink and SomaScan platforms in 3936 Chinese adults**


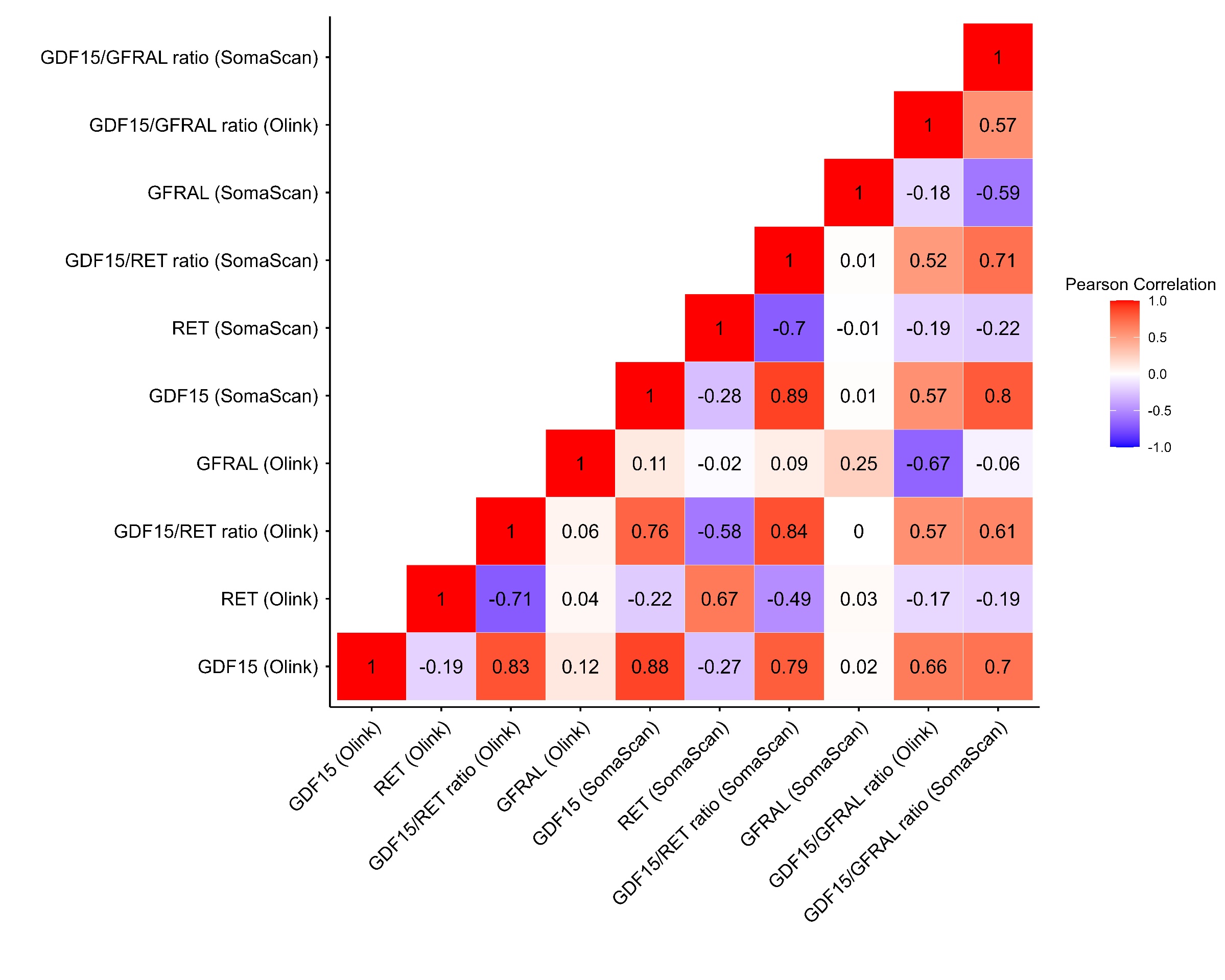


# **Supplementary Figure 2. Associations of age with plasma levels of GDF15, RET, GFRAL and their ratios measured by Olink and SomaScan by smoking status in 1555 male Chinese adults**

Former smokers (n=265) were excluded. Estimated marginal means (95% CI) for each age category among 1101 current and 454 never-regular smokers were obtained from a linear model adjusted for study area (10 groups), fasting time, ambient temperature (linear and squared terms), plate ID (Olink analyses only), and case-subcohort ascertainment. The length of the y-axis represents approximately 3 standard deviations of the corresponding plasma protein concentration. NPX values correspond to Olink measurements and RFU values to SomaScan measurements.

Abbreviations: CI=confidence interval; GDF15=growth/differentiation factor 15; GFRAL=GDNF family receptor alpha-like; NPX=normalised protein expression; RET=proto-oncogene tyrosine-protein kinase receptor Ret; RFU=relative fluorescence units.

**Olink**

**e) GDF15/GFRAL ratio**

**d) GFRAL**

**c) GDF/RET ratio**

**b) RET**

**a) GDF15**


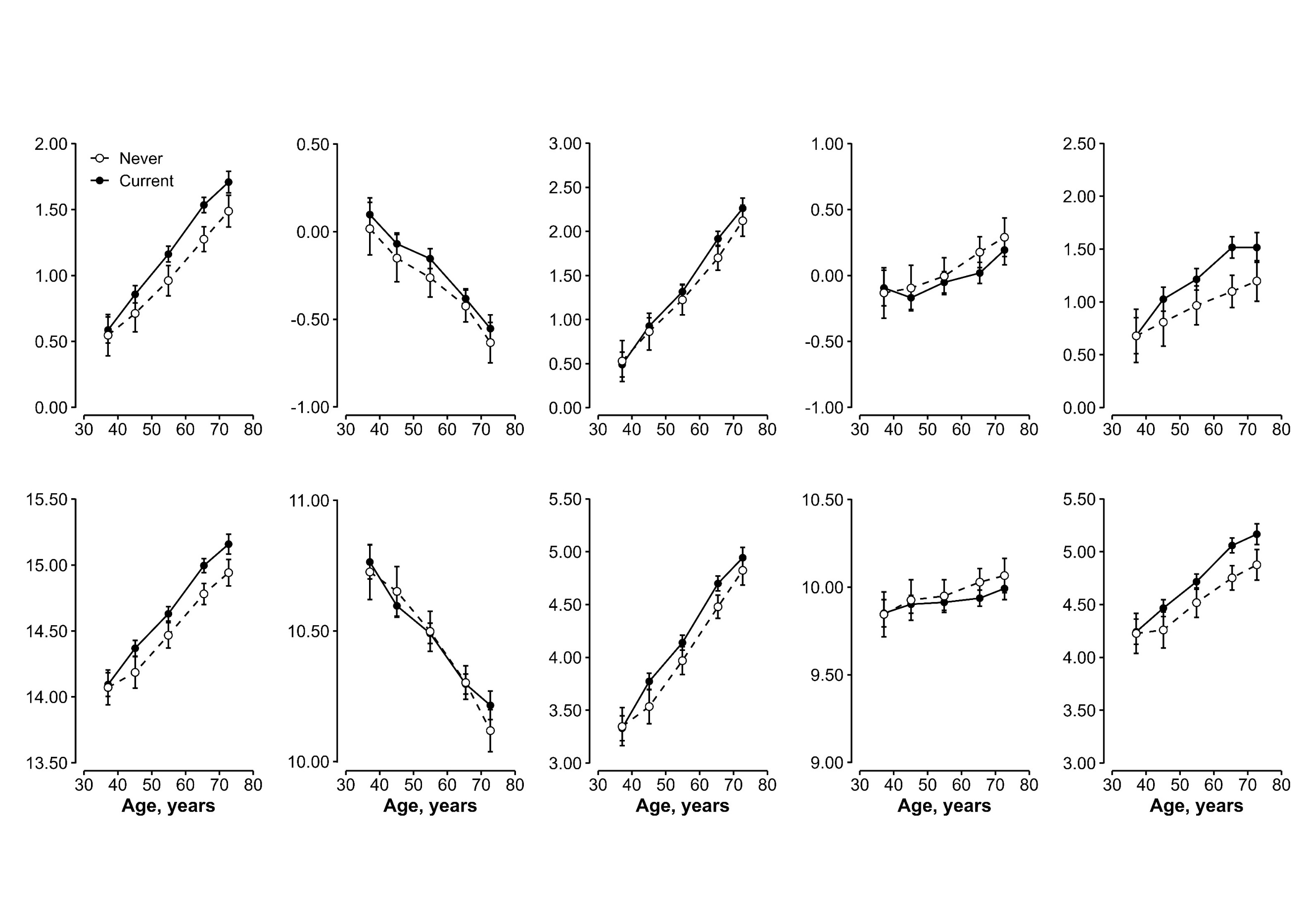


**Mean protein levels, NPX/RFU**

**SomaScan**

# **Supplementary Figure 3. Associations of age with plasma levels of GDF15, RET, GFRAL and their ratios measured by Olink and SomaScan by smoking status in 2083 female Chinese adults**

Former smokers (n=33) were excluded. Estimated marginal means (95% CI) for each age category among 86 current and 1997 never-regular smokers were obtained from a linear model adjusted for study area (10 groups), fasting time, ambient temperature (linear and squared terms), plate ID (Olink analyses only), and case-subcohort ascertainment. The length of the y-axis represents approximately 3 standard deviations of the corresponding plasma protein concentration. NPX values correspond to Olink measurements and RFU values to SomaScan measurements.

Abbreviations: CI=confidence interval; GDF15=growth/differentiation factor 15; GFRAL=GDNF family receptor alpha-like; NPX=normalised protein expression; RET=proto-oncogene tyrosine-protein kinase receptor Ret; RFU=relative fluorescence units.

**Olink**

**e) GDF15/GFRAL ratio**

**d) GFRAL**

**c) GDF/RET ratio**

**b) RET**

**a) GDF15**


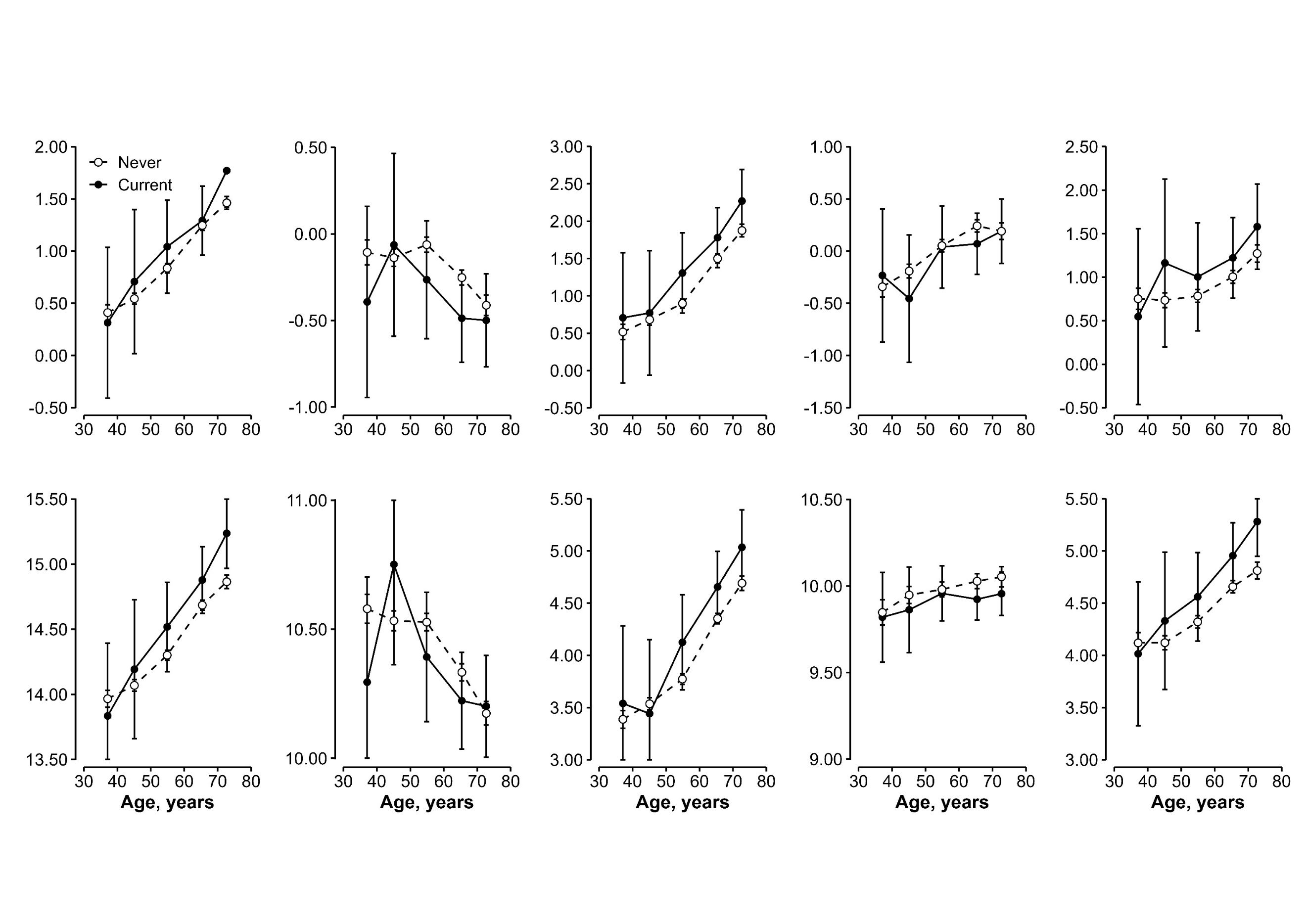


**Mean protein levels, NPX/RFU**

**SomaScan**

# **Supplementary Figure 4. Associations of plasma levels of GDF15, RET, GFRAL and their ratios measured by Olink and SomaScan with BMI, by smoking status in 1555 Chinese males**

Former smokers (n=265) were excluded. Estimated marginal means (95% CI) for each protein fifth among 1101 current and 454 never-regular smokers were obtained from a linear model adjusted for age (linear and squared terms), study area (10 groups), fasting time, ambient temperature (linear and squared terms), plate ID (Olink analyses only), and case-subcohort ascertainment. The length of the y-axis represents approximately 2 standard deviations of BMI. NPX values correspond to Olink measurements and RFU values to SomaScan measurements.

Abbreviations: BMI=body mass index; CI=confidence interval, GDF15=growth/differentiation factor 15; GFRAL=GDNF family receptor alpha-like; NPX=normalised protein expression; RET=proto-oncogene tyrosine-protein kinase receptor Ret; RFU=relative fluorescence units.

**Olink**


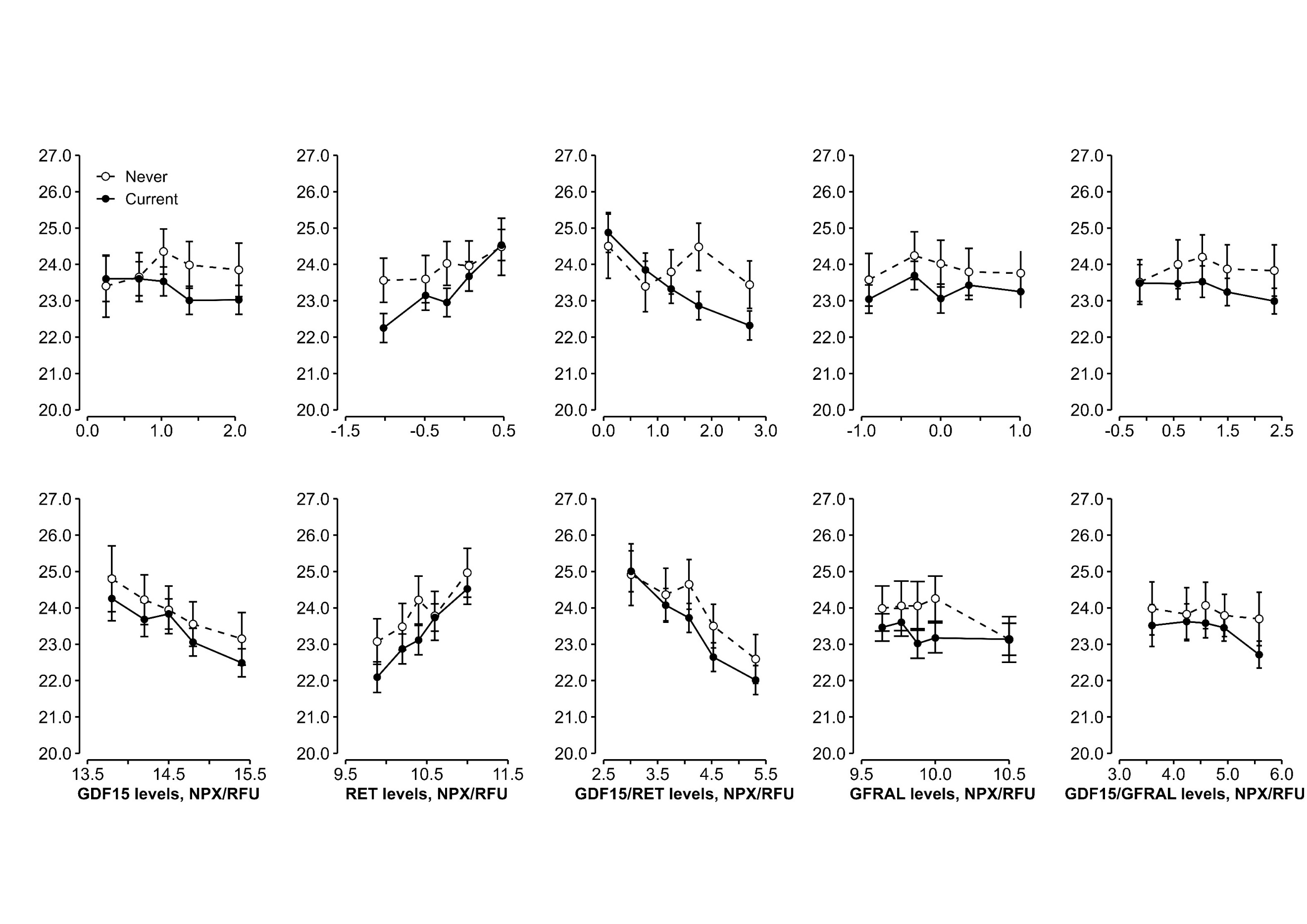


**e) GDF15/GFRAL ratio**

**d) GFRAL**

**c) GDF/RET ratio**

**b) RET**

**a) GDF15**

**SomaScan**

**Mean BMI, kg/m^2^**

# **Supplementary Figure 5. Associations of plasma levels of GDF15, RET, GFRAL and their ratios measured by Olink and SomaScan with BMI, by smoking status in 2083 Chinese females**

Former smokers (n=33) were excluded. Estimated marginal means (95% CI) for each protein fifth among 86 current and 1997 never-regular smokers were obtained from a linear model adjusted for age (linear and squared terms), study area (10 groups), fasting time, ambient temperature (linear and squared terms), plate ID (Olink analyses only), and case-subcohort ascertainment. The length of the y-axis represents approximately 2 standard deviations of BMI. NPX values correspond to Olink measurements and RFU values to SomaScan measurements.

Abbreviations: BMI=body mass index; CI=confidence interval, GDF15=growth/differentiation factor 15; GFRAL=GDNF family receptor alpha-like; NPX=normalised protein expression; RET=proto-oncogene tyrosine-protein kinase receptor Ret; RFU=relative fluorescence units.

**e) GDF15/GFRAL ratio**

**d) GFRAL**

**c) GDF/RET ratio**

**Olink**

**b) RET**

**a) GDF15**

**
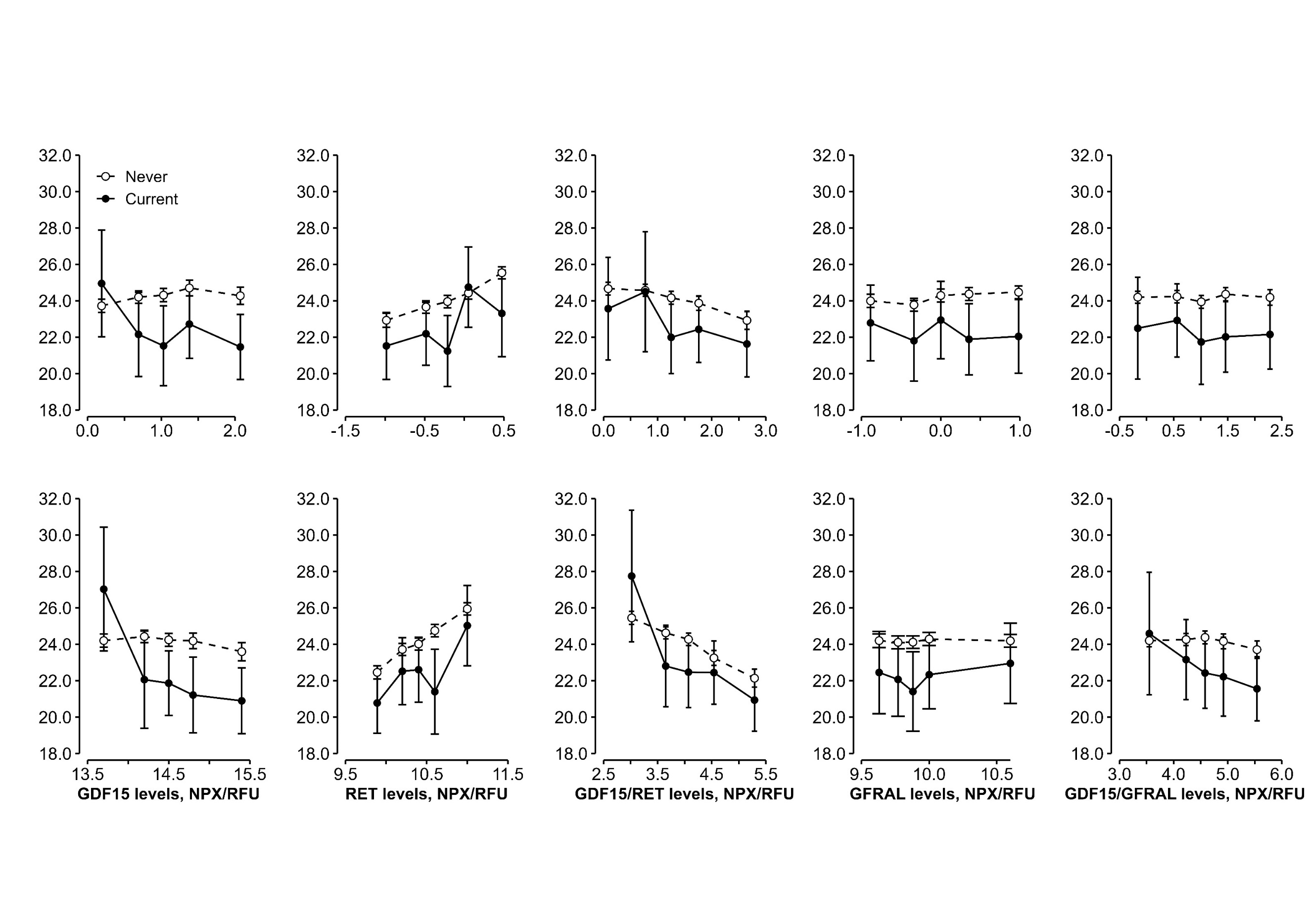
**

**SomaScan**

**Mean BMI, kg/m^2^**

# **Supplementary Figure 6. Associations of plasma levels of GDF15, RET and GFRAL and their ratios measured by Olink and Somascan, with WC by smoking status in 3638 Chinese adults**

Former smokers (n=298) were excluded. Estimated marginal means (95% CI) for each protein fifth among 1187 current and 2451 never-regular smokers were obtained from a linear model adjusted for age (linear and squared terms), sex, study area (10 groups), fasting time, ambient temperature (linear and squared terms), plate ID (Olink analyses only), and case-subcohort ascertainment. The length of the y-axis represents approximately 2 standard deviations of WC. NPX values correspond to Olink measurements and RFU values to SomaScan measurements.

Abbreviations: CI=confidence interval, GDF15=growth/differentiation factor 15; GFRAL=GDNF family receptor alpha-like; NPX=normalised protein expression; RET=proto-oncogene tyrosine-protein kinase receptor Ret; RFU=relative fluorescence units; WC=waist circumference.

**Olink**

**e) GDF15/GFRAL ratio**

**d) GFRAL**

**c) GDF/RET ratio**

**b) RET**

**a) GDF15**


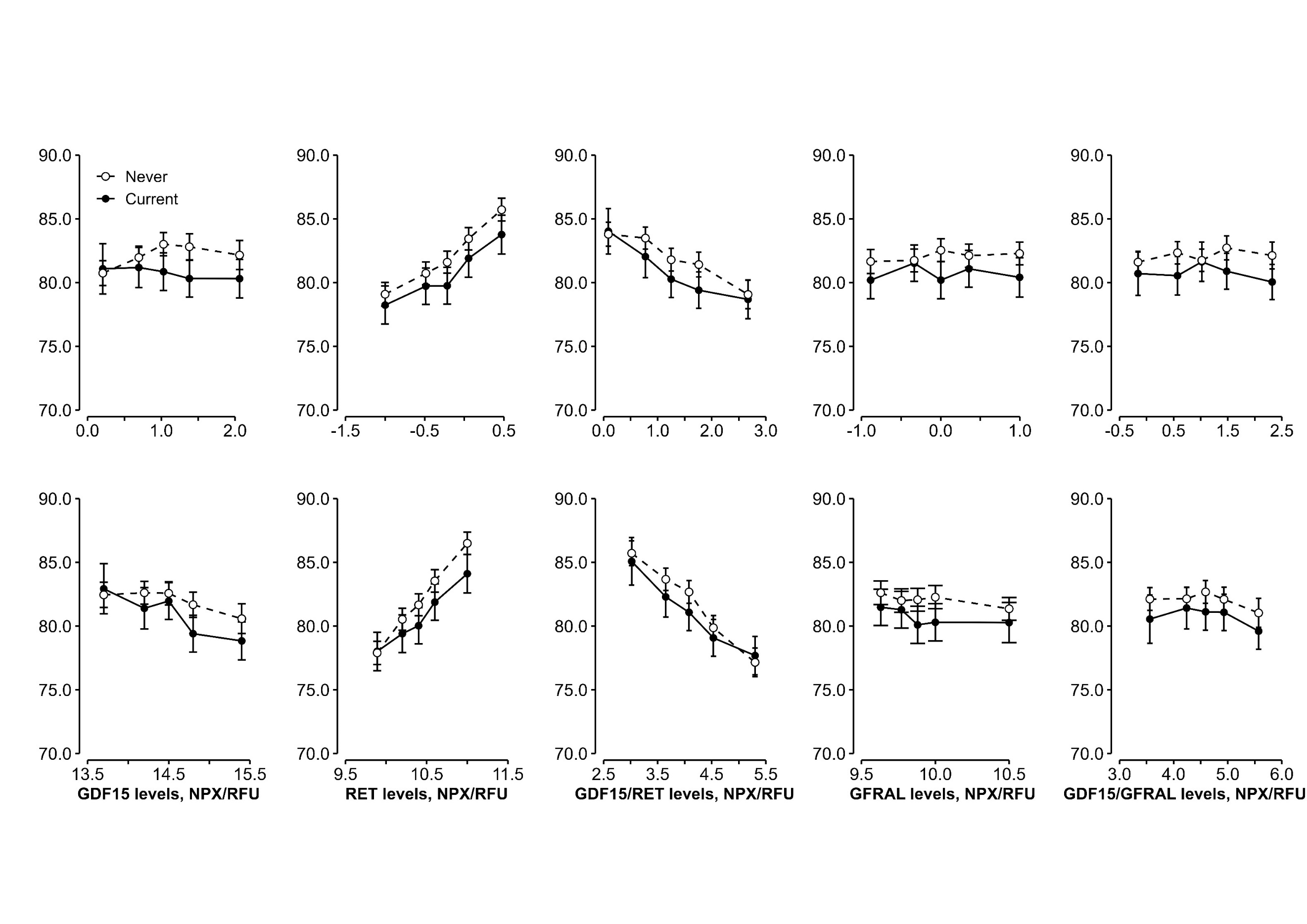


**SomaScan**

**Mean waist circumference, cm**

# **Supplementary Figure 7. Associations of plasma levels of GDF15, RET, GFRAL and their ratios measured by Olink and SomaScan with HC, by smoking status in 3638 Chinese adults**

Former smokers (n=298) were excluded. Estimated marginal means (95% CI) for each protein fifth among 1187 current and 2451 never-regular smokers were obtained from a linear model adjusted for age (linear and squared terms), sex, study area (10 groups), fasting time, ambient temperature (linear and squared terms), plate ID (Olink analyses only), and case-subcohort ascertainment. The length of the y-axis represents approximately 2 standard deviations of HC. NPX values correspond to Olink measurements and RFU values to SomaScan measurements.

Abbreviations: CI=confidence interval, GDF15=growth/differentiation factor 15; GFRAL=GDNF family receptor alpha-like; HC=hip circumference; NPX=normalised protein expression; RET=proto-oncogene tyrosine-protein kinase receptor Ret; RFU=relative fluorescence units.

**Olink**

**d) GFRAL**

**c) GDF/RET ratio**

**b) RET**

**a) GDF15**

**e) GDF15/GFRAL ratio**


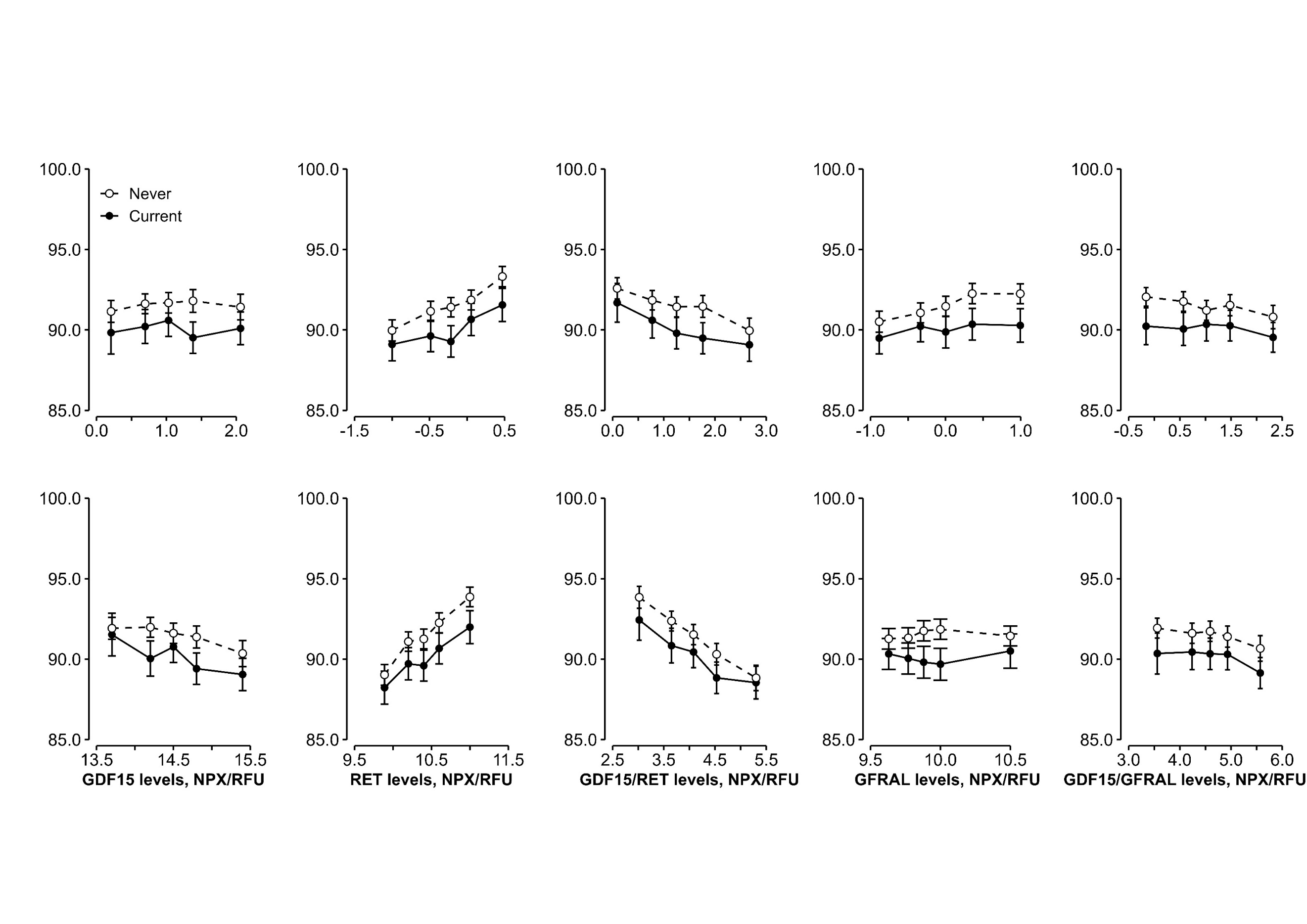


**SomaScan**

**Mean hip circumference, cm**

# **Supplementary Figure 8. Associations of plasma levels of GDF15, RET, GFRAL and their ratios measured by Olink and SomaScan with WHR, by smoking status in 3638 Chinese adults**

Former smokers (n=298) were excluded. Estimated marginal means (95% CI) for each protein fifth among 1187 current and 2451 never-regular smokers were obtained from a linear model adjusted for age (linear and squared terms), sex, study area (10 groups), fasting time, ambient temperature (linear and squared terms), plate ID (Olink analyses only), and case-subcohort ascertainment. The length of the y-axis represents approximately 2 standard deviations of WHR. NPX values correspond to Olink measurements and RFU values to SomaScan measurements.

Abbreviations: CI=confidence interval, GDF15=growth/differentiation factor 15; GFRAL=GDNF family receptor alpha-like; NPX=normalised protein expression; RET=proto-oncogene tyrosine-protein kinase receptor Ret; RFU=relative fluorescence units; WHR=waist/hip ratio.

**Olink**

**e) GDF15/GFRAL ratio**

**c) GDF/RET ratio**

**b) RET**

**a) GDF15**

**d) GFRAL**


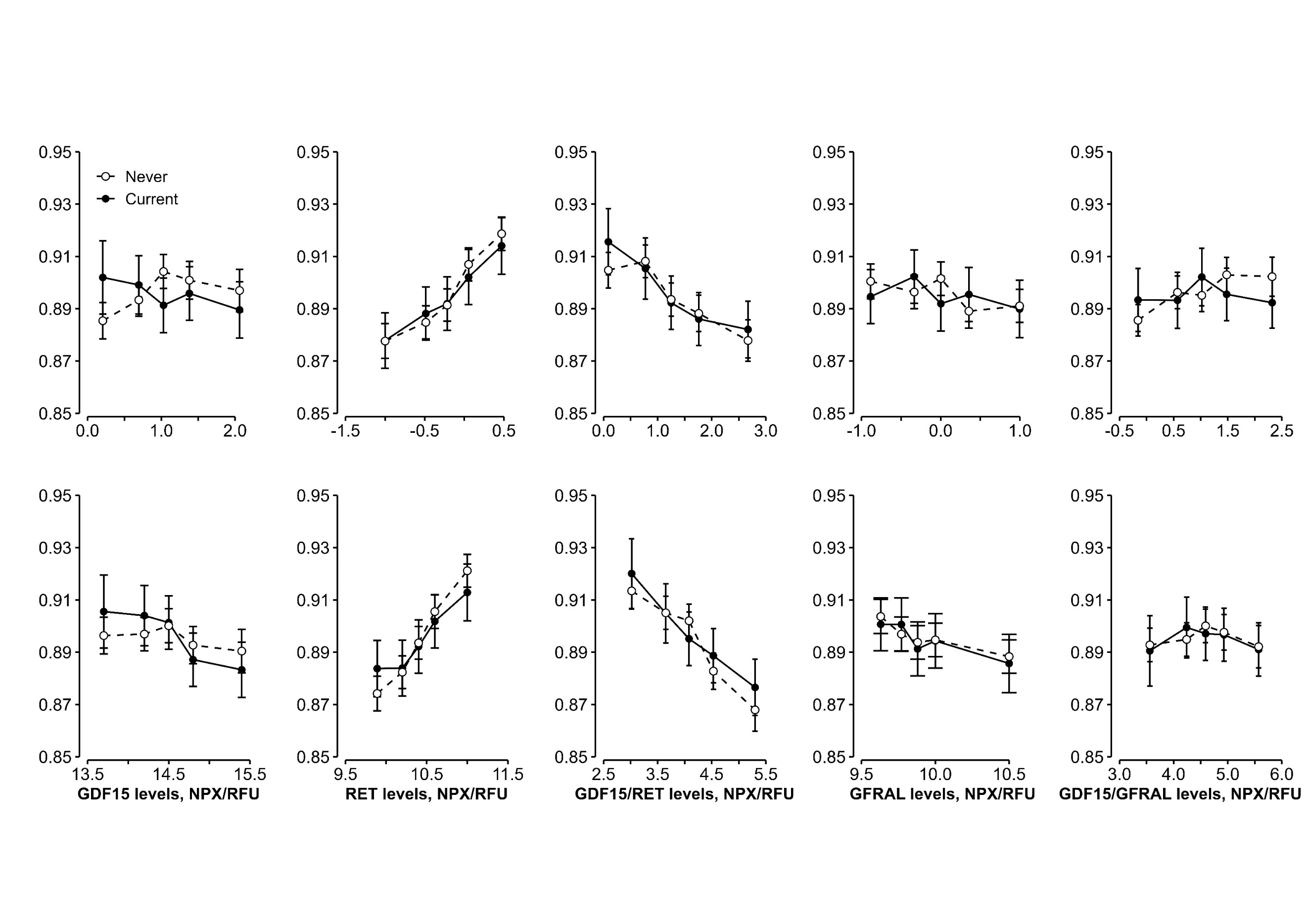


**SomaScan**

**Mean waist/hip ratio**

# **Supplementary Figure 9. Associations of plasma levels of GDF15, RET, GFRAL and their ratios measured by Olink and SomaScan with BF%, by smoking status in 3634 Chinese adults**

Former smokers (n=298) were excluded, and data is available for 3934 individuals for BF%. Estimated marginal means (95% CI) for each protein fifth among 1185 current and 2449 never-regular smokers were obtained from a linear model adjusted for age (linear and squared terms), sex, study area (10 groups), fasting time, ambient temperature (linear and squared terms), plate ID (Olink analyses only), and case-subcohort ascertainment. The length of the y-axis represents approximately 2 standard deviations of BF%. NPX values correspond to Olink measurements and RFU values to SomaScan measurements.

Abbreviations: BF%=body fat percentage; CI=confidence interval, GDF15=growth/differentiation factor 15; GFRAL=GDNF family receptor alpha-like; NPX=normalised protein expression; RET=proto-oncogene tyrosine-protein kinase receptor Ret; RFU=relative fluorescence units.

**Olink**

**e) GDF15/GFRAL ratio**

**d) GFRAL**

**c) GDF/RET ratio**

**b) RET**

**a) GDF15**


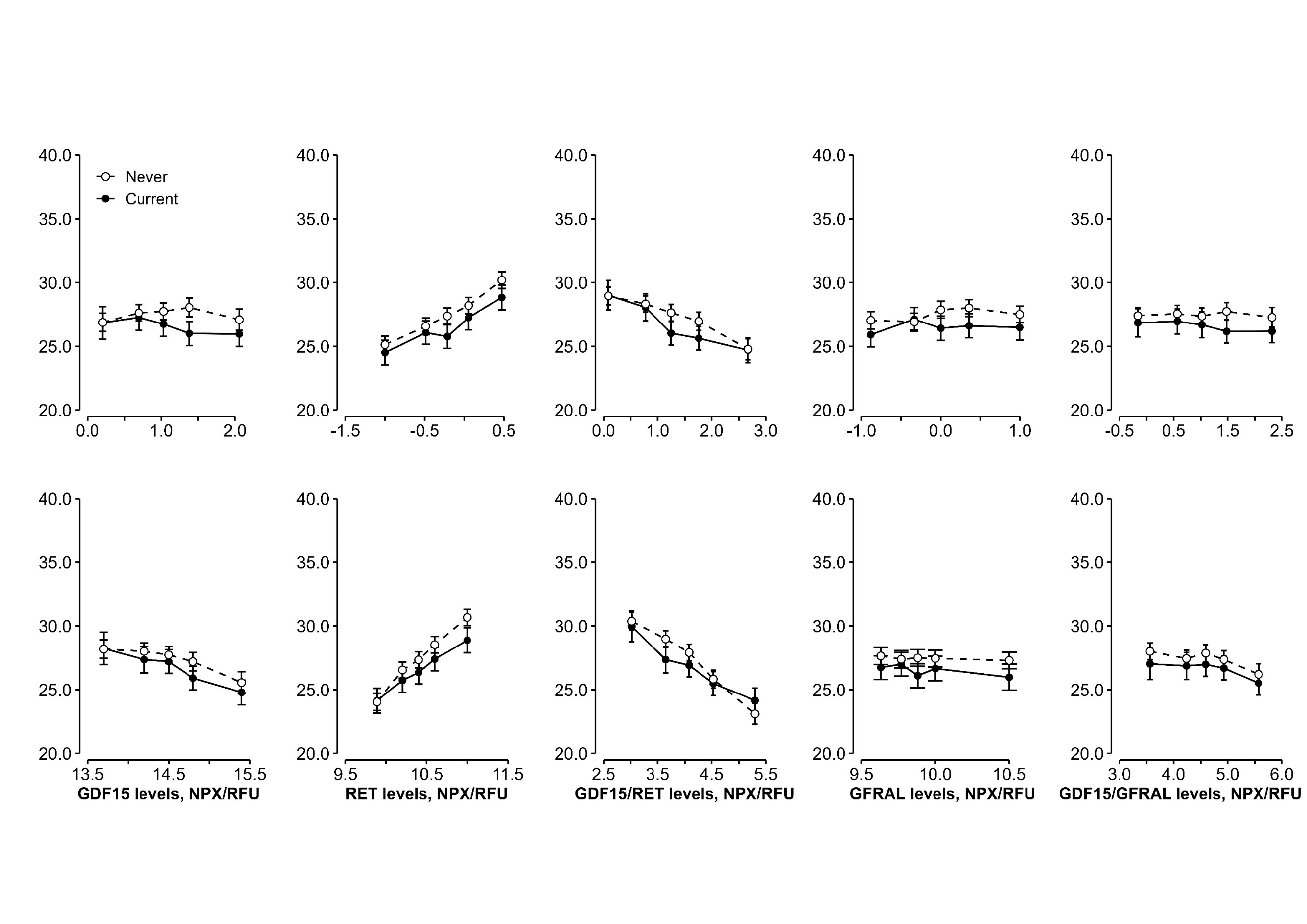


**SomaScan**

**Mean body fat percentage, %**

# **Supplementary Figure 10. Associations of smoking intensity with plasma levels of RET measured by Olink and SomaScan in 3638 Chinese adults**

Former smokers (n=298) were excluded. Estimated marginal means (95% CI) for each smoking category among 1187 current and 2451 never-regular smokers were obtained from a linear model adjusted for age (linear and squared terms), sex, study area (10 groups), fasting time, ambient temperature (linear and squared terms), plate ID (Olink analyses only), BMI, alcohol, physical activity, hypertension status, kidney disease status and case-subcohort ascertainment. Tests for trend include only current smokers. NPX values correspond to Olink measurements and RFU values to SomaScan measurements.

Abbreviations: NPX=normalised protein expression; RET=proto-oncogene tyrosine-protein kinase receptor Ret; RDU=relative fluorescence units.


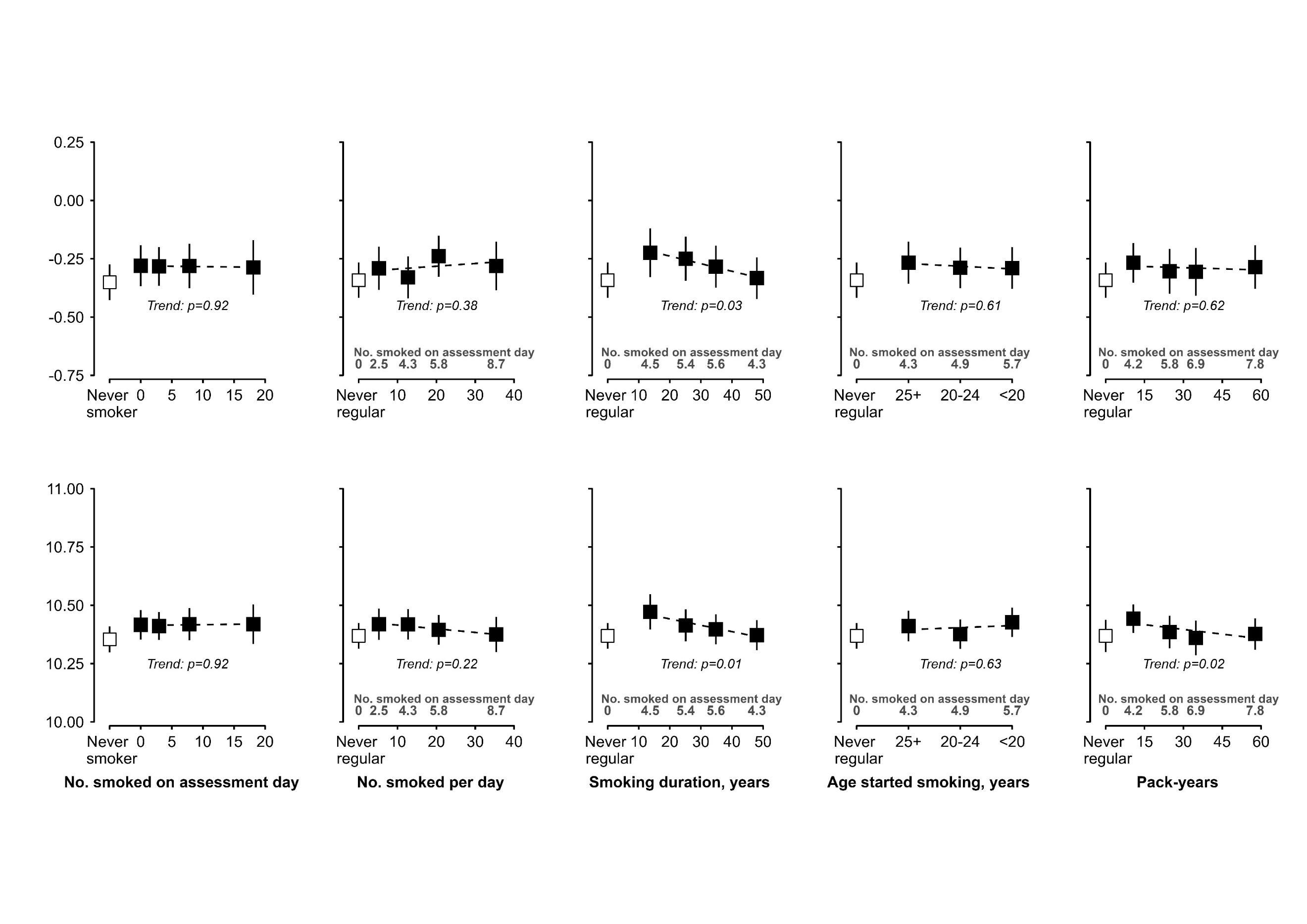


**SomaScan**

**Olink**

**Mean RET levels, NPX/RFU**

# **Supplementary Figure 11. Associations of smoking intensity with plasma levels of the GDF15/RET ratio measured by Olink and SomaScan in 3638 Chinese adults**

Former smokers (n=298) were excluded. Estimated marginal means (95% CI) for each smoking category among 1187 current and 2451 never-regular smokers were obtained from a linear model adjusted for age (linear and squared terms), sex, study area (10 groups), fasting time, ambient temperature (linear and squared terms), plate ID (Olink analyses only), BMI, alcohol, physical activity, hypertension status, kidney disease status and case-subcohort ascertainment. Tests for trend include only current smokers. NPX values correspond to Olink measurements and RFU values to SomaScan measurements.

Abbreviations: GDF15=growth/differentiation factor 15; NPX=normalised protein expression; RET=proto-oncogene tyrosine-protein kinase receptor Ret; RDU=relative fluorescence units.


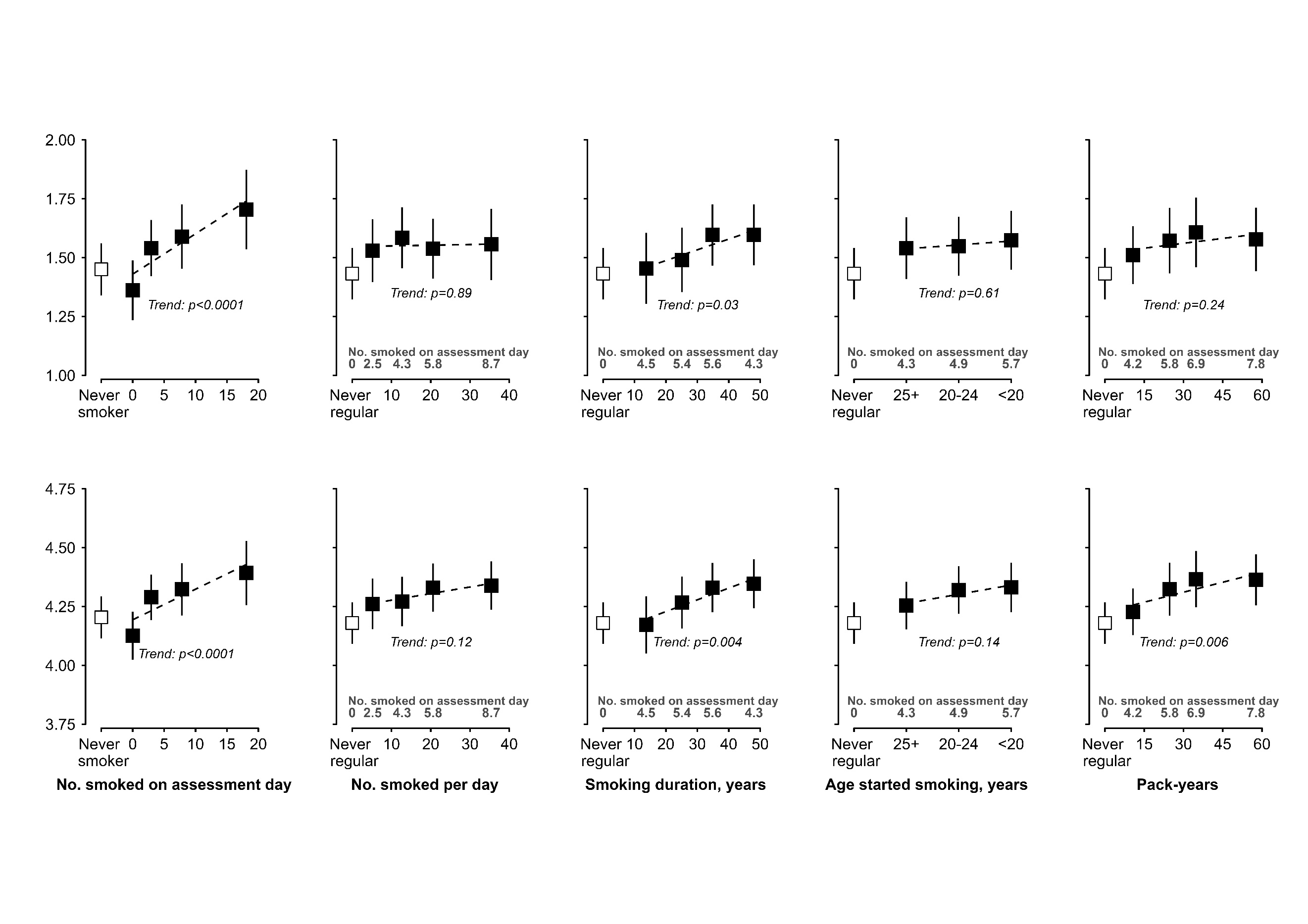


**Olink**

**SomaScan**

**Mean GDF15/RET levels, NPX/RFU**

# **Supplementary Figure 12. Associations of smoking intensity with plasma levels of GFRAL measured by Olink and Somascan in 3638 Chinese adults**

Former smokers (n=298) were excluded. Estimated marginal means (95% CI) for each smoking category among 1187 current and 2451 never-regular smokers were obtained from a linear model adjusted for age (linear and squared terms), sex, study area (10 groups), fasting time, ambient temperature (linear and squared terms), plate ID (Olink analyses only), BMI, alcohol, physical activity, hypertension status, kidney disease status and case-subcohort ascertainment. Tests for trend include only current smokers. NPX values correspond to Olink measurements and RFU values to SomaScan measurements.

Abbreviations: GFRAL=GDNF family receptor alpha; NPX=normalised protein expression; RDU=relative fluorescence units.


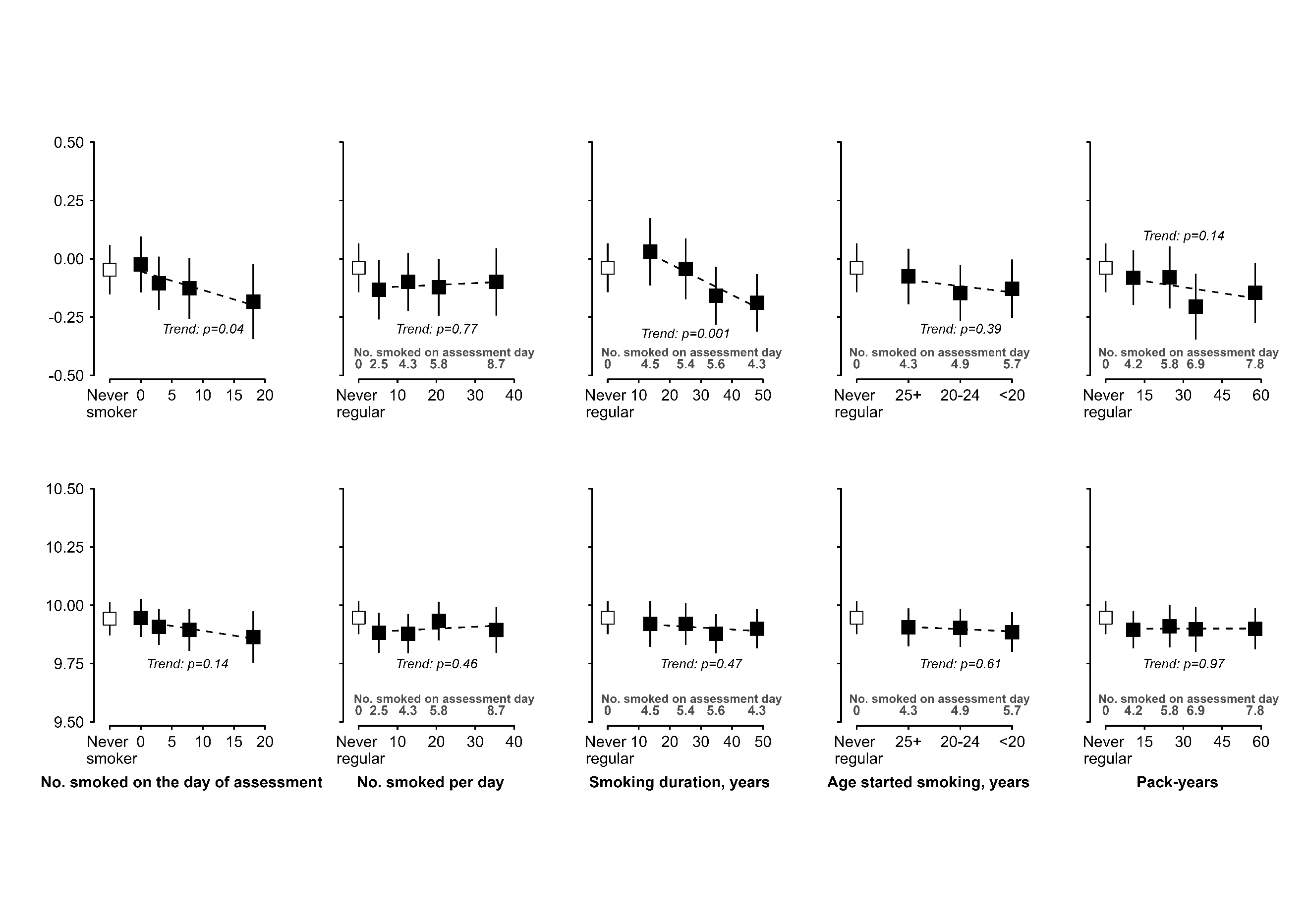


**Olink**

**SomaScan**

**Mean GFRAL levels, NPX/RFU**

# **Supplementary Figure 13. Associations of smoking intensity with plasma levels of the GDF15/GFRAL ratio measured by Olink and SomaScan in 3638 Chinese adults**

Former smokers (n=298) were excluded. Estimated marginal means (95% CI) for each smoking category among 1187 current and 2451 never-regular smokers were obtained from a linear model adjusted for age (linear and squared terms), sex, study area (10 groups), fasting time, ambient temperature (linear and squared terms), plate ID (Olink analyses only), BMI, alcohol, physical activity, hypertension status, kidney disease status and case-subcohort ascertainment. Tests for trend include only current smokers. NPX values correspond to Olink measurements and RFU values to SomaScan measurements.

Abbreviations: GDF15=growth/differentiation factor 15; GFRAL=GDNF family receptor alpha; NPX=normalised protein expression; RDU=relative fluorescence units.


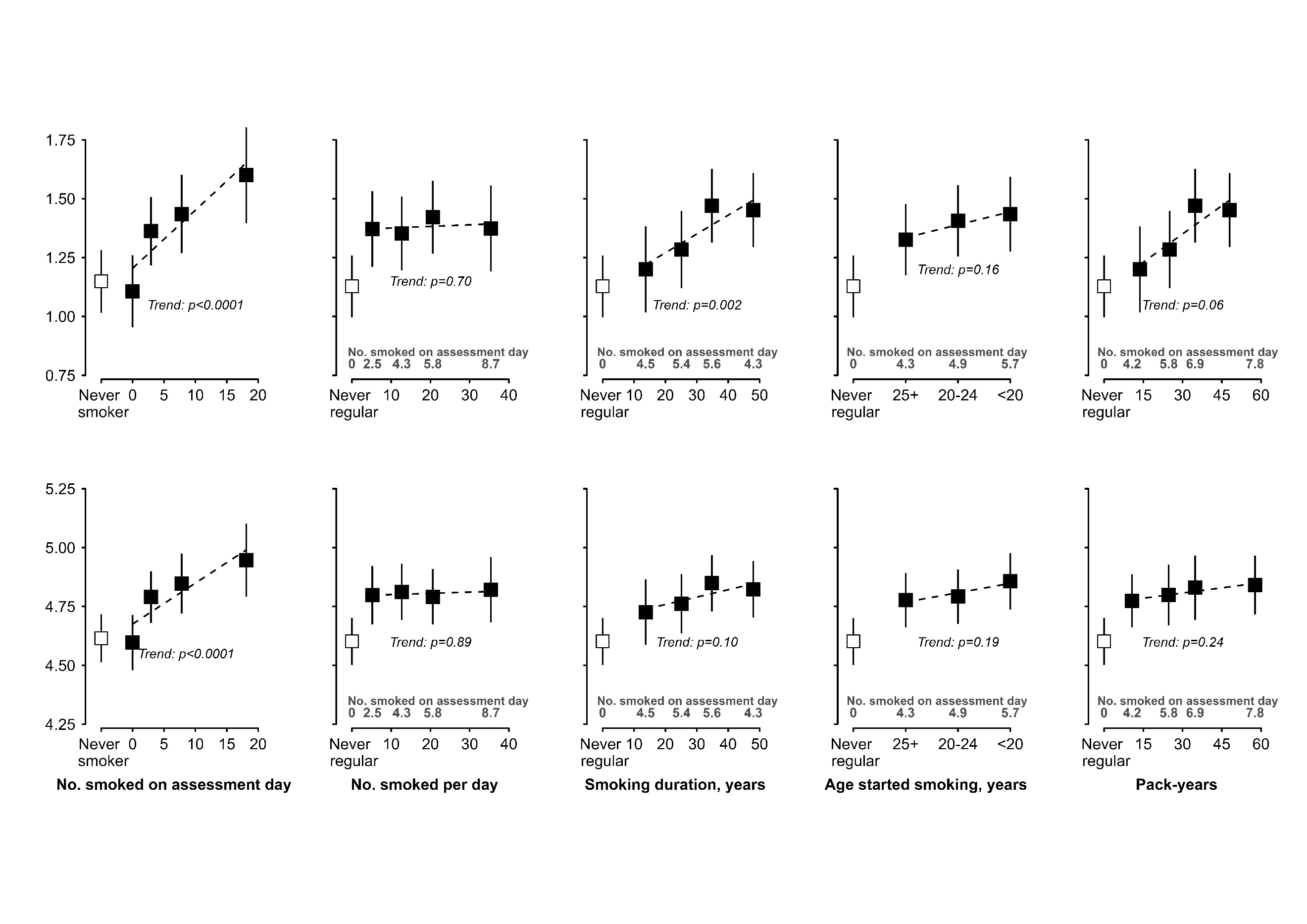


**Olink**

**SomaScan**

**Mean GDF15/GFRAL levels, NPX/RFU**

# **Supplementary Figure 14. Associations of years since smoking cessation and plasma levels of GDF15, GDF15/RET and GDF15/GFRAL measured by Olink and SomaScan in 1485 Chinese adults**

Never-regular smokers (n=2451) were excluded. Estimated marginal means (95% CI) for each smoking category among 298 former and 1187 current-regular smokers were obtained from a linear model adjusted for age (linear and squared terms), sex, study area (10 groups), fasting time, ambient temperature (linear and squared terms), plate ID (Olink analyses only), BMI, alcohol, physical activity, hypertension status, kidney disease status and case-subcohort ascertainment. Tests for trend include only former smokers. NPX values correspond to Olink measurements and RFU values to SomaScan measurements.

Abbreviations: GDF15=growth/differentiation factor 15; GFRAL=GDNF family receptor alpha; NPX=normalised protein expression; RET= proto-oncogene tyrosine-protein kinase receptor Ret; RDU=relative fluorescence units.

**Olink**

**c) GDF15/GFRAL ratio**

**b) GDF15/RET ratio**

**a) GDF15**


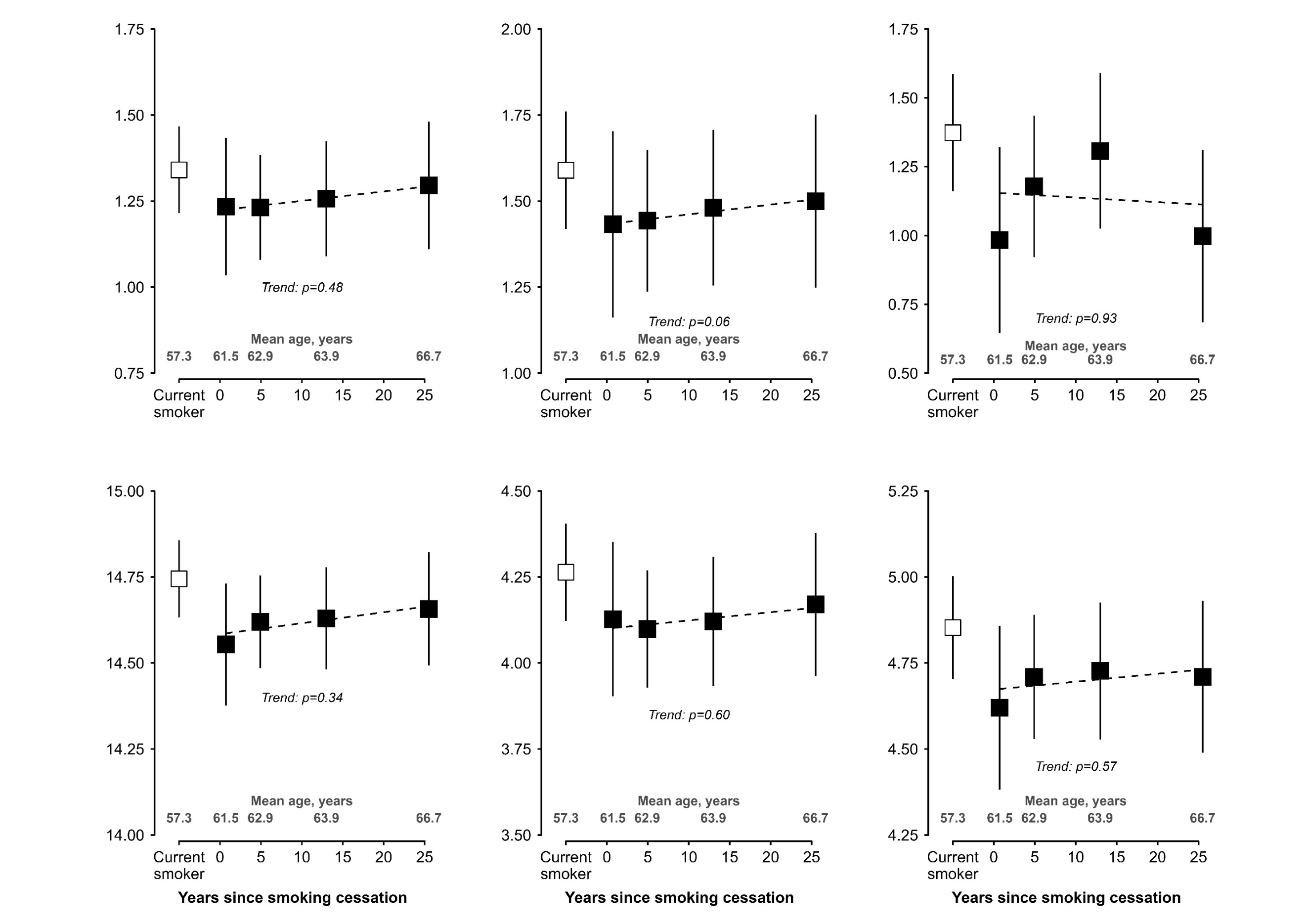


**Mean protein levels, NPX/RFU**

**SomaScan**
